# NAD(H)-dependent corepressor CTBP1 integrates metabolic signals to drive oncogenic programs

**DOI:** 10.3389/fimmu.2026.1797122

**Published:** 2026-03-27

**Authors:** Enes Yağız Akdaş, Kirill Schneider, Dingyu Lu, Khouloud Hachani, Erika Lynn Roberts, Mohamad Ghanem, Ali Bashiri Dezfouli, Barbara Wollenberg

**Affiliations:** Department of Otolaryngology, Head and Neck Surgery, School of Medicine, Technical University of Munich (TUM), Munich, Germany

**Keywords:** cancer metabolism and redox regulation, CTBP1, epigenetic control of oncogenic programs, NAD(H)-dependent transcriptional corepression, tumor immune modulation, tumor progression and therapy resistance

## Abstract

C-terminal binding protein 1 (CTBP1) is an NAD(H)-dependent transcriptional corepressor that links cellular metabolic state to chromatin-based gene regulation. Since its initial identification, CTBP1 has been implicated in a broad range of developmental and pathological processes, including cancer. Increasing evidence suggests that CTBP1 integrates redox cues, epigenetic repression, and transcriptional networks controlling epithelial plasticity, survival, DNA damage responses, and therapy resistance across multiple malignancies. In this review, we critically examine the current literature on CTBP1 structure, regulation, and function, with a particular focus on cancer biology. We synthesize findings from experimental studies that support CTBP1 as an oncogenic driver in specific tumor contexts, while explicitly highlighting settings in which evidence remains indirect, incomplete, or confined to locus-associated noncoding RNAs rather than CTBP1 protein itself. We further discuss the constraints and uncertainties surrounding CTBP1-directed therapeutic strategies, including incomplete mechanistic validation, context-dependent functions, and potential on-target toxicity arising from CTBP1’s roles in normal metabolic and transcriptional homeostasis. Overall, this review positions CTBP1 as a context-dependent transcriptional integrator whose pathological functions are shaped by metabolic stress and epigenetic regulation, while emphasizing unresolved questions and experimental gaps that must be addressed before CTBP1 can be reliably evaluated as a therapeutic target.

## 1 Introduction

C-terminal binding protein 1 (CTBP1) is an NAD(H)-dependent transcriptional corepressor that functions as a metabolic sensor linking cellular redox state to epigenetic gene regulation (1). Through preferential binding to NADH and redox-dependent dimerization, CTBP1 assembles multiprotein repression complexes at PLDLS motif-containing transcription factors, recruiting chromatin-modifying enzymes such as HDACs and histone methyltransferases to silence gene expression (2). This biochemical property enables CTBP1 to couple high glycolytic flux and elevated NADH/NAD+ ratios to durable transcriptional repression programs.

In cancer, this metabolic-epigenetic integration places CTBP1 at a strategic node controlling epithelial plasticity, apoptosis, DNA damage responses, stem-like states, immune evasion, and therapy resistance. However, the strength and specificity of evidence vary substantially across tumor types. In some malignancies, CTBP1 protein-level perturbation (genetic KD, CRISPR editing, or pharmacologic inhibition) demonstrates clear oncogenic dependency. In others, functional data derive primarily from CTBP-family-level analyses that do not disentangle CTBP1 from its paralog CTBP2, or from regulatory outputs encoded at the CTBP1 locus (e.g., *CTBP1-DT*/*CTBP1-AS* transcripts), rather than from CTBP1 protein itself.

This review critically evaluates CTBP1 structure, biochemical regulation, physiological functions, and tumor-specific roles, with explicit distinction between CTBP1-specific mechanisms, CTBP-family-level evidence, and locus-associated noncoding RNA biology. By systematically stratifying mechanistic strength and translational validation across cancer types, we aim to define where CTBP1 represents a bona fide oncogenic driver, where evidence remains indirect or conflated with CTBP2, and what experimental gaps must be addressed before CTBP1 can be reliably advanced as a therapeutic target.

## 2 Structural and biochemical properties of CTBP1: NAD(H) sensing and domain architecture

**Figure 1:**
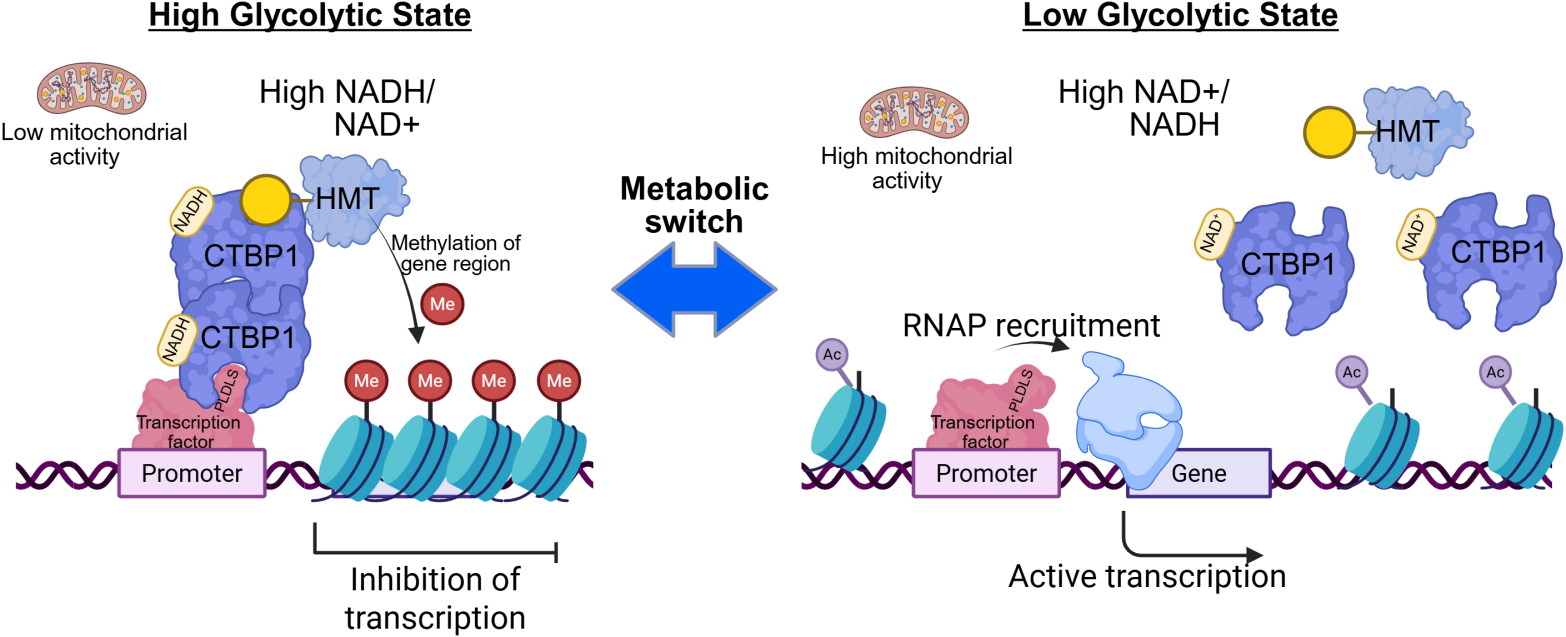
Metabolic control of CTBP1-dependent transcriptional repression. Schematic model illustrating the redox-dependent switch in CTBP1 function. Under high-glycolytic conditions with elevated NADH levels (left), NADH promotes CTBP1 dimerization. Dimeric CTBP1 is recruited to PLDLS-containing transcription factors at target gene promoters, where it scaffolds repressive chromatin-modifying complexes such as histone methyltransferase (HMT). This leads to histone methylation (Me), chromatin compaction, and stable transcriptional repression. In contrast, under low-glycolytic conditions with a high NAD+/NADH ratio (right), CTBP1 predominantly exists in a monomeric state. Loss of stable CTBP1 dimers impairs scaffolding of repressive complexes, leading to preservation of histone acetylation (Ac). Open chromatin allows RNA polymerase II (RNAP) recruitment and results in transcriptional activation of genes. This reversible metabolic switch links cellular redox state to epigenetic regulation and gene expression programs, leading to the adjustment of gene expression to the metabolic state of the cell.

C-terminal binding protein 1 (CTBP1) is an NAD(H)-dependent transcriptional corepressor that links the cellular metabolic state to gene expression (3). It was originally identified as a PLDLS motif-binding protein at the C-terminus of the adenovirus E1A protein (4, 5) and later shown to act broadly as a repressor of tumor suppressors, apoptotic, and pro-differentiation genes (6–11). Structurally, CTBP1 harbors a Rossmann-fold dehydrogenase-like domain known to bind NADH with much higher affinity than NAD+ (12). NADH binding promotes CTBP1 dimerization and stabilization of repression complexes, whereas oxidation (high NAD+) favors monomeric CTBP1 with reduced repressive function (13). Thus, CTBP1 acts as a redox sensor: in high glycolytic flux (elevated NADH/NAD+ ratio) it promotes dimer formation and gene repression, while oxidative conditions reverse this effect (14, 15). This metabolically controlled switch is depicted schematically below (**Figure 1**), where NADH-driven CTBP1 dimerization facilitates the recruitment of chromatin-modifying complexes and epigenetic silencing, whereas NAD+-driven CTBP1 monomers fail to sustain transcriptional repression.

## 3 Expression, structure, and regulation of CTBP1

**Figure 2:**
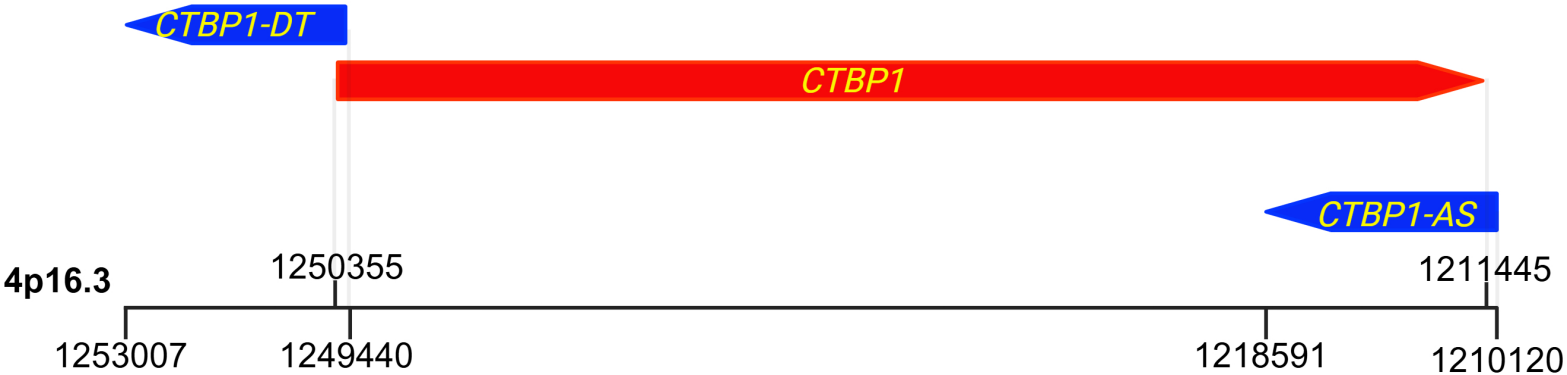
Genomic organization of the CTBP1 locus and associated transcripts. Schematic representation of the *CTBP1* locus on human chromosome 4p16.3 (GRCh38) illustrating the relative positions and transcriptional orientations of the protein-coding *CTBP1* gene and its associated long noncoding RNAs. *CTBP1* (red; chr4:1,211,434-1,250,333, reverse strand) is transcribed from the reverse (−) strand. *CTBP1-DT* (blue, left; chr4:1,248,877-1,288,856, forward strand) represents a divergent long noncoding RNA transcribed independently from the forward (+) strand upstream of *CTBP1*, whereas *CTBP1-AS* (blue, right; chr4:1,210,120-1,218,591, forward strand) is a natural antisense lncRNA transcribed from the forward (+) strand overlapping the *CTBP1* locus. Arrows indicate the direction of transcription; vertical lines and the scale bar denote approximate genomic boundaries. All three transcripts are independently initiated and do not arise from alternative splicing or processing of the *CTBP1* mRNA.

The *CTBP1* gene (located on chr4p16.3) is transcribed from the reverse strand and produces mRNA encoding CTBP1 protein (16, 17). Although no alternative transcription start sites have been reported, alternative splicing generates 2 isoforms: *CTBP1-long* (*CTBP1-L*) and *CTBP1-short* (*CTBP1-S*) which differ by 13 N-terminal amino acids (12, 18). *CTBP1* gene is also known to transcribe 2 long noncoding RNAs (lncRNA), known as *CTBP1-Antisense* (*CTBP1-AS*, also known as *PCAT10)* (19), and *CTBP1-DT (*also known as *CTBP1-AS1*, or *CTBP1-AS2)* (20). Physiological roles of *CTBP1*-*AS* and *CTBP1-DT* remain incompletely defined; however, both transcripts have been implicated in post-transcriptional regulation in several cancers (19–21). Specifically, in prostate cancer, *CTBP1-AS* has been reported to recruit the PSF/HDAC repressive complex to the *CTBP1* promoter, resulting in transcriptional repression of *CTBP1* (19). Beyond this context, however, direct evidence demonstrating effects of these lncRNAs on CTBP1 protein abundance, localization, or activity remains limited. The transcriptional orientations of *CTBP1*, *CTBP1*-*DT*, and *CTBP1*-*AS* are shown in **Figure 2**.

**Figure 3:**
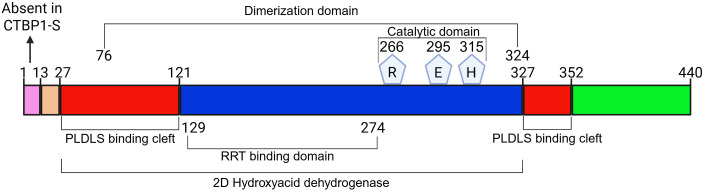
Schematic domain organization of CTBP1 and its functional regions. The diagram illustrates the linear architecture of CTBP1. The N-terminus (1–13aa) exists only in CTBP1 long isoform and is lacking in CTBP1 short (CTBP1-S) isoform. The central region (blue) represents the conserved dehydrogenase-like core with a Rossmann-fold responsible for high-affinity NADH binding, which governs CTBP1 dimerization (76–324aa) and redox-sensitive corepressor activity. Highlighted within this core are key amino acids involved in enzymatic homology (R, E, H). Flanking regions shown in red denote the repression-associated interaction domains (generates one domain in 3D structure). The C-terminal region (green) contains motifs important for CTBP1’s regulation and some other protein bindings. Brackets indicate approximate boundaries of functional domains.

CTBP1 comprises multiple overlapping functional regions (**Figure 3**):

- the PLDLS-binding substrate cleft (approx. residues 13–121 together with approx. residues 252–327) (2, 4),
- the dimerization interface (residues ~76–324) (22),
- the Rossmann-like NAD(H)-binding pocket (residues 181–204) (3, 13),
- the RRT-recognition surface (residues 129–274) (2),
- the D-2-hydroxyacid dehydrogenase (D2-HDH)-like catalytic core, which covers most of the protein (roughly residues 27–352), with a catalytic triad formed by R266, E295, and H315 (3, 13).

The N-terminal residues 1–13 are unique to the CTBP1-L isoform, generated by alternative splicing, and are absent in CTBP1-S (23). Isoform-specific antibodies raised against this unique N-terminal segment show overlapping nuclear-cytoplasmic distributions of CTBP1-L and CTBP1-S as well as contribute similarly to transcriptional regulation and membrane-trafficking-related functions in neurons; showing that the 1–13 aa extension does not significantly alter basic corepressor activity (12, 24). Functionally, CTBP1-L-specific 13 aa are best viewed as an isoform-defining regulatory segment, distinguishing the full-length nuclear corepressor. CTBP1-S, the N-terminally truncated form of CTBP1-L, is found to exert specialized roles in the Golgi (25).

Deletion of the RRT binding region does not change the repressor activity of CTBP1 (2). In contrast, PLDLS binding cleft mutations directly affect its cofactor binding mechanisms (26). CTBP1 binds to PLDLS motifs on DNA-binding repressors (a conserved function of CTBP family proteins). Under certain conditions, when the PLDLS-binding cleft is occupied by a cofactor, the RRT binding region is also used as a hinge to retain bi-motif binding proteins (2). This mechanism explains how CTBP1 coordinates histone modifications when it is bound to a transcription factor (2). Bound CTBP1 attracts chromatin remodelers; notably histone deacetylases (HDAC1/2) and histone methyltransferases (HMTs) (27). Through these, CTBP1 induces repressive histone marks at target promoters.

The dehydrogenase domain binds NAD(H) but has minimal enzymatic activity; instead, NADH or NAD+ binding allosterically controls CTBP1 oligomerization. Crystal and biochemical studies show CTBP1 has >100-fold higher affinity for NADH than NAD+, establishing it as an NADH sensor (1, 28). NADH binding favors CTBP1 dimer/tetramer assembly, which stabilizes interactions with chromatin-modifying enzymes (22, 28, 29). In contrast, oxidative conditions (e.g. antioxidants, low glycolysis) raise NAD+, shifting CTBP1 towards monomers that dissociate from DNA (30, 31). For example, the antioxidant Tempol lowers cellular NADH levels and causes CTBP1 to disengage from the BRCA1 promoter in squamous carcinoma cells (30). In summary, CTBP1 is an NADH/NAD+-dependent transcriptional corepressor that fine-tunes gene expression according to the metabolic (redox) state.

## 4 Cellular functions of CTBP1

CTBP1 has multiple, context-dependent functions that are closely linked to its subcellular localization and to the cell’s metabolic (redox) state. In the nucleus, it regulates development, metabolism, and cell survival. Genetic studies in mice underscore its developmental functions. *CTBP1* KO mice are viable but smaller and have reduced survival, whereas its paralog *CTBP2* KO mice die *in utero*; double *CTBP1*/*CTBP2*-null embryos show markedly earlier lethality than either single KO (8). This indicates a partial redundancy: *CTBP1* and *CTBP2* share many targets, but *CTBP2* is more critical for embryogenesis.

Metabolically, CTBP1 functions as a glucose- and redox-sensitive transcriptional regulator in several tissues. In polycystic ovary syndrome, CTBP1 is upregulated in granulosa cells and its expression correlates with disturbed hormone and lipid profiles. Mechanistically, CTBP1 represses estrogen synthesis, as well as dampens lipogenic gene expression and reduces lipid storage. Through these pathways, CTBP1 links elevated NADH and insulin resistance to altered steroidogenesis and lipid homeostasis. Additional studies implicate CTBP1 in adipocyte dysfunction, browning, and oxidative-stress responses, underscoring a broader role in systemic energy balance and redox biology. Importantly, CTBP1 maintains mitochondrial integrity under normoglycemia; it represses pro-apoptotic mitochondrial genes (e.g., *BAX*, *PUMA*) when glucose (and NADH) is sufficient (32). In *CTBP1*-null cells, *BAX* is derepressed, mitochondria become fragmented, and respiratory function declines (32). Therefore, CTBP1 couples the energy state to cell survival by silencing mitochondrial apoptosis programs. Similarly, CTBP1 can prevent excessive anoikis (detachment-induced apoptosis) in tissues by repressing fibronectin inhibitors (33).

Although CTBP1 is predominantly characterized as a transcriptional corepressor, there is evidence that it can, in specific contexts, act as a transcriptional co-activator (34, 35). For example, CTBP1 directly interacts with TCF4 and augments the expression of β-catenin target genes, including *CD44*, *SNAI1L*, *C-MYC*, and *LGR5* (36, 37).

Beyond its nuclear functions, CTBP1 also exerts important cytoplasmic roles in membrane trafficking and synaptic function. At the Golgi, CTBP1-S isoform acts as a brefeldin-A-sensitive fission factor, and acyl-CoA-dependent lysophosphatidic-acid acyltransferase. Depletion or catalytic inhibition of CTBP1-S blocks the fission of post-Golgi carriers and COPI vesicles, leading to elongated Golgi tubules and impaired cargo export (25, 38, 39). CTBP1-S thereby sits at the core of a fission machinery with ARF1, PLD and protein kinase D that dynamically tunes Golgi architecture to secretory demand (38, 40). In neurons, CTBP1 is highly enriched at presynaptic terminals, where it is anchored to the active-zone scaffold proteins Bassoon and Piccolo (24, 41–43). Activity-dependent shuttling between presynapse and nucleus allows CTBP1 to couple synaptic performance to gene expression programs (41, 42). Functionally, loss or KD of *CTBP1* alters synapse density, changes synaptic vesicle pool size and distribution, and slows compensatory endocytosis after exocytosis, consistent with a role for CTBP1-mediated membrane fission and PLD1 activation in synaptic vesicle recycling (41, 44). Thus, cytoplasmic CTBP1 links its classical transcriptional corepressor activity to the control of Golgi and presynaptic membrane remodeling, integrating secretory and synaptic output with cellular activity and metabolic state.

Besides its physiological roles, CTBP1 functions as an oncogenic transcriptional regulator in several cancer contexts. It forms a repressor complex with p300 and FOXO3a to silence pro-apoptotic genes, including *BAX*, *BIM*, *BIK*, and *PMAIP1*, thereby promoting cell survival (45, 46). CTBP1 also downregulates the death receptors *DR4* and *DR5* genes, further supporting resistance to apoptosis (47). By repressing tumor suppressors such as *PTEN* and *APC*, CTBP1 modulates Wnt/β-catenin signaling and enhances proliferation, migration, and invasion (48, 49).

**Figure 4:**
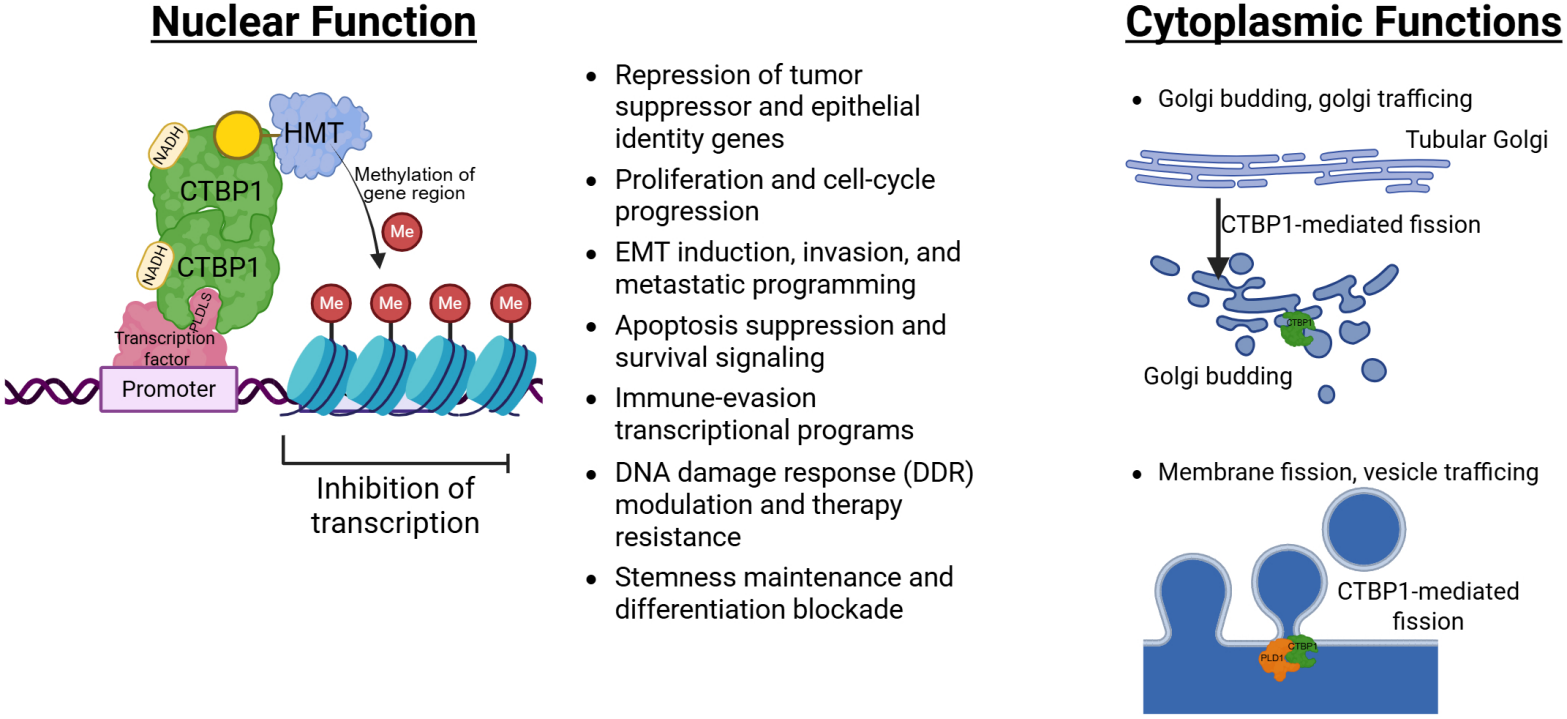
Schematic overview illustrating the compartment-specific activities of CTBP1. Left panel (nucleus): Under metabolically reduced conditions with elevated NADH, CTBP1 binds NADH and forms dimers that are recruited to gene promoters via PLDLS-containing transcription factors. Dimeric CTBP1 acts as a transcriptional corepressor by scaffolding chromatin-modifying enzymes, leading to the deposition of repressive histone marks, chromatin compaction, and transcriptional silencing of target genes. Right panel (cytoplasm): Cytoplasmic CTBP1, predominantly represented by the short isoform (CTBP1-S), localizes to the Golgi apparatus, where it participates in membrane remodeling and fission events required for post-Golgi vesicle formation. Through interactions with lipid-modifying and trafficking machinery, CTBP1 regulates vesicle scission and membrane dynamics, thereby linking cellular metabolic state to secretory and trafficking pathways. Together, this dual localization enables CTBP1 to integrate metabolic cues with both transcriptional repression in the nucleus and membrane dynamics in the cytoplasm. CTBP1: green, PLD1: orange, Me: methyl, HMT: Histone methyltransferase.

Collectively, CTBP1’s physiological roles span embryonic development, metabolic homeostasis, mitochondrial health, and membrane dynamics, highlighting how its dysregulation can drive diverse disease states, including cancer. The major nuclear and cytoplasmic functions of CTBP1 are schematically summarized in **Figure 4**.

## 5 CTBP1 in cancer biology

**Figure 5:**
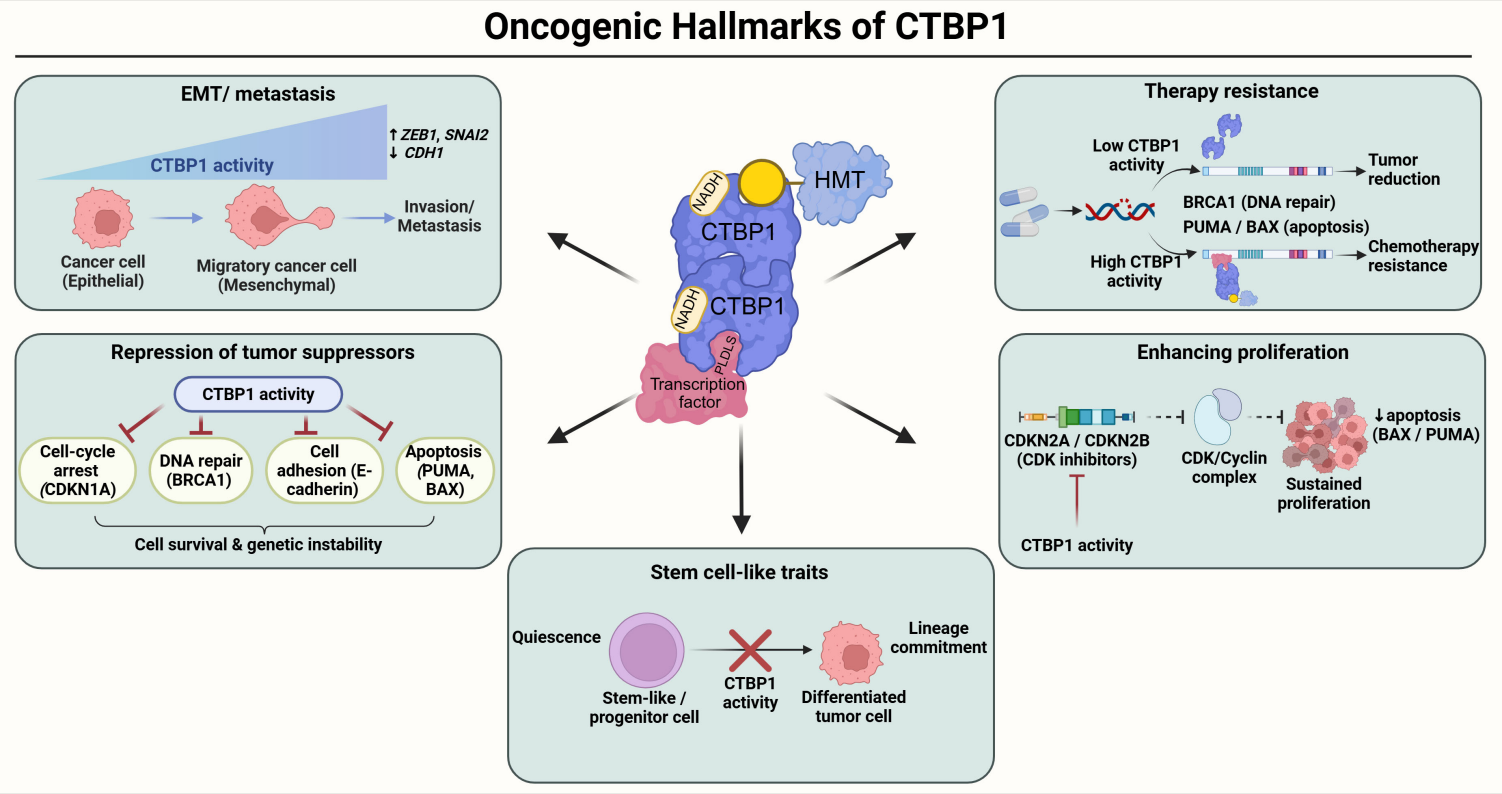
CTBP1 in cancer biology. Schematic illustration summarizing the major oncogenic functions of CTBP1 across diverse malignancies. CTBP1 is frequently overexpressed or hyperactivated and acts as an NADH-dependent transcriptional corepressor by forming dimers and being recruited to chromatin via PLDLS-containing transcription factors, assembling repressive complexes that include histone-modifying enzymes. Elevated CTBP1 activity promotes repression of tumor suppressor genes, including *CDH1* (E-cadherin), *BRCA1*, *CDKN1A* (p21), and the pro-apoptotic factors *PUMA* and *BAX*, thereby enhancing cell survival and genetic instability. CTBP1 further drives EMT and metastasis by repressing epithelial programs and indirectly favoring mesenchymal regulators such as *ZEB1* and *SNAI2*. In parallel, CTBP1 enhances proliferation by suppressing cyclin-dependent kinase inhibitors (*CDKN2A*, *CDKN2B*) and apoptotic pathways, enabling sustained CDK–cyclin activity. CTBP1-mediated repression of DNA repair and apoptotic genes further contributes to therapy resistance, whereas reduced CTBP1 activity restores DNA damage responses and apoptosis, leading to tumor reduction. In addition, CTBP1 supports stem cell-like traits by inhibiting lineage commitment and differentiation programs, maintaining quiescent, progenitor-like tumor cell states. Together, these processes illustrate how CTBP1 integrates metabolic and epigenetic regulation to promote tumor progression, aggressiveness, and poor clinical outcomes.

CTBP1 is frequently overexpressed or hyperactivated and functions as an oncogenic transcriptional corepressor across diverse malignancies, including breast cancer (15, 50, 51), melanoma (52), prostate (53, 54), lung adenocarcinoma (49), hepatocellular carcinoma (55), gastric cancer (56), endometrial carcinoma (57), glioma (58), pancreatic ductal adenocarcinoma (59), leukemia (60), head and neck squamous cell carcinoma (30) and others. Elevated CTBP1 generally associates with loss of tumor-suppressor expression, enhanced proliferation, invasion, therapy resistance, and/or poor clinical outcome. The principal oncogenic hallmarks of CTBP1 are schematically depicted below in **Figure 5**.

Repression of tumor suppressors: CTBP1 silences genes like *CDH1* (E-cadherin), *BRCA1*, *CDKN1A* (p21), *PUMA*, *BAX*, and others, promoting cell survival and genetic instability (9, 30, 32, 50, 52, 61).

Promotion of EMT and invasion: By repressing epithelial markers (*CDH1* and other epithelial transcription factors) and inducing repressors (*ZEB1*, *SNAI2* indirectly), CTBP1 fosters epithelial-to-mesenchymal transition (EMT) and metastasis (9, 62, 63).

Enhancing proliferation: CTBP1 stimulates cell cycle progression and stemness by repressing cyclin-dependent kinase inhibitors (*CDKN2A*, *CDKN2B*) and apoptosis effectors (52, 64).

Chemotherapy resistance: CTBP1-mediated repression of DNA repair genes (like *BRCA1*) and pro-apoptotic factors can confer resistance to genotoxic drugs (46). Conversely, *CTBP1* KD or its chemical inhibition sensitizes cells to chemotherapy and radiation by restoring DNA-damage responses (9, 14, 30, 46, 65).

Stem cell-like traits: *CTBP1* overexpression increases tumor stem-like populations by inhibiting differentiation programs. For instance, CTBP1 activation expands basal progenitors in mammary tissues and maintains quiescence in cancer stem cells (9, 50, 60, 66).

Below we review the role of *CTBP1* gene in specific cancers, highlighting targets, pathways, and clinical implications. A structured comparative overview of CTBP1-related evidence across tumor types is provided in **Table 1**, integrating the nature of protein-level perturbation (e.g., KD, CRISPR-mediated KO, overexpression, interaction disruption), availability of *in vivo* validation, presence of locus-associated lncRNA mechanisms (e.g., CTBP1-DT/CTBP1-AS), pharmacologic or dimerization-based targeting strategies, and degree of clinical correlation. This framework allows direct comparison of mechanistic depth, isoform specificity, and translational readiness across malignancies.

**Table 1:** Evidence stratification of CTBP1 across tumor types.

Tumor type	Protein-level perturbation (KD/KO/CRISPR/OE)	*In vivo* models	lncRNA evidence	Inhibitor/drug evidence	Clinical correlation	Evidence strength	Key ref
Breast cancer	KD, OE	high-fat-diet mouse model	*CTBP1-AS CTBP1-DT*	Dimerization inhibitors (non-selective CTBP)	Yes	Strong protein-level mechanistic	(9, 35, 46, 51, 67, 75)
Prostate cancer	KD, mutational perturbation	high-fat-diet mouse model	*CTBP1-AS*	No selective CTBP1 inhibitor	Yes	Strong protein-level mechanistic	(19, 78, 123, 124)
Colorectal cancer	KD, pharmacologic inhibition	CRC xenograft growth	*CTBP1-DT*	MTOB, NSC95397 (CTBP1/2 dual)	Yes	Strong protein-level mechanistic	(36, 81, 84, 85, 127, 128)
Lung cancer (NSCLC)	KD	patient-derived tumor xenografts syngeneic mouse experiments	*CTBP1-DT*	Cell-penetrating PLDLS-motif peptides	Yes	Moderate protein-level	(49, 87, 112)
Glioma	KD	No	*CTBP1-DT*	No selective CTBP1 drug	Limited	Mixed, mostly mechanistic-complex	(20, 58, 89)
AML/Leukemia	interaction disruption; CTBP-family	Limited (xenotransplant)	No clear driver	PLDLS-competitor constructs	Limited	Weak- CTBP family	(90)
HNSCC	promoter binding/redox-modulation; no KO/KD	No	*CTBP1-DT*	No selective inhibitor	Yes	Moderate protein-level	(30, 97)
Ovarian cancer	CTBP-family KD/Inhibition	No	*CTBP1-DT*	CTBP family dimerization linked to platinum response	Limited	Weak- CTBP family	(14, 47, 100)
ESCC	CRISPR KO	No	Not studied	No selective inhibitor	Limited	Strong protein-level	(98, 102, 103, 138)
Osteosarcoma	KD, complex-based interaction studies	No	Not studied	No selective inhibitor	Limited	Moderate mechanistic	(45, 104, 105, 135)
Melanoma	OE, pharmacologic inhibition	melanoma animal model	Not studied	Selective CTBP1 inhibitor	Yes	Strong + pharmacologic validation	(52, 106, 107, 109)
PDAC	CTBP1/2 combined	mutant KRAS-driven PDAC mouse model	*CTBP1-DT*	No CTBP1-specific	Yes	Weak- CTBP1 family	(59, 110, 120)
HCC	KD	No	*CTBP1-DT*	No selective inhibitor	Yes	Strong protein-level	(55, 63, 139)
Gastric cancer	CRISPR KO	*In vivo* metastasis models	*CTBP1-DT*	No selective inhibitor	Yes	Strong protein-level	(56, 114–116)
Cervical cancer	No protein data	No	*CTBP1-DT*	No inhibitor data	Yes	lncRNA-only	(117)
ccRCC	No protein data	No	*CTBP1-DT*	No inhibitor data	Yes	lncRNA-only	(118)
Cholangiocarcinoma	No protein data	No	*CTBP1-DT*	No inhibitor data	Limited	lncRNA-only	(120)

Summary of protein-level perturbation (KD, KO, CRISPR, overexpression (OE), or interaction disruption), availability of *in vivo* validation, locus-associated lncRNA evidence (*CTBP1-DT*/*CTBP1-AS*), pharmacologic targeting approaches, clinical correlation, and overall qualitative evidence strength. “Strong” denotes direct genetic validation with functional support; “Moderate” indicates mechanistic evidence without full *in vivo* or isoform resolution; “Weak-CTBP family” reflects CTBP-complex or combined CTBP1/2 studies; “lncRNA-only” indicates absence of direct CTBP1 protein-level interrogation.

### 5.1 CTBP1 in breast cancer

In breast carcinoma, CTBP1 functions as a redox-responsive corepressor that couples metabolic inputs to EMT, metastasis, and therapy resistance. Zhao et al. showed that CTBP1 forms a complex with ZEB1 at the *SREBF2* promoter in human breast cancer cells, repressing *SREBF2* (the master regulator of cholesterol biosynthesis), lowering intracellular cholesterol, stabilizing TGF-β receptors, and amplifying TGF-β signaling; this axis contributes EMT, invasion, and metastatic dissemination in orthotopic breast cancer models (67).

Metabolic-syndrome models further link CTBP1 hyperactivation to obesity-driven breast cancer progression. In high-fat-diet mice with metabolic syndrome, De Luca et al. found that a lowered NAD^+^/NADH ratio increases CTBP1 activity and expression in mammary tumors, reprograms mRNA/miRNA networks controlling proliferation, EMT, mammary development and cell-to-cell communication, and accelerates tumor growth (68). A follow-up study by Farré et al. showed that CTBP1 and metabolic syndrome cooperatively induce a pro-metastatic mRNA/miRNA signature, increase circulating tumor cells and distant metastases, whereas CTBP1 depletion largely abrogates these effects, underscoring CTBP1 as a key mediator of metabolic-syndrome-associated breast cancer aggressiveness (66).

Therapy-resistant, stem-like breast cancer cells also depend on CTBPs dimerization. Banerjee et al. generated metformin-resistant breast cancer cells with stem-like features and showed that their survival requires NADH-dependent CTBPs dimerization; small-molecule inhibitors that disrupt CTBPs dimerization selectively kill the metformin-resistant population and restore metformin sensitivity (69). In a related study, Kreuzer et al. demonstrated that enhanced glycolysis promotes CTBPs dimerization, sustaining hypoxia-induced CAIX expression and the survival of stem-like breast cancer cells; genetic or pharmacologic CTBPs inhibition suppresses CAIX and depletes this aggressive subpopulation (70). Consistently, CAIX^high^ circulating tumor cells mark stem-like, metastasis-prone breast cancer cells and represent a potential therapeutic target in advanced disease (70).

At the level of transcriptional control, CTBP1 directly represses key tumor-suppressor genes in breast epithelial cells. Deng et al. showed that CTBP1 occupies the *BRCA1* and *CDH1* (E-cadherin) promoters in breast cancer cells; CTBP1 overexpression or enforced recruitment reduces *BRCA1* and *CDH1* expression, contributing to defective DNA repair and loss of epithelial adhesion (9). In primary human cells, CTBP1 represses the cyclin-dependent kinase inhibitor *CDKN2A*, thereby facilitating cell-cycle progression, a mechanism considered relevant to breast tumorigenesis (71). Post-transcriptionally, the miR-644a/CTBP1/p53 axis modulates chemoresistance. Raza et al. showed that miR-644a down-regulates *CTBP1*, shifts p53 towards pro-apoptotic targets (e.g., *PUMA*, *NOXA*) and simultaneously suppresses survival and EMT programmes, thereby reversing multi-drug resistance in breast cancer models (46). Together, these data place CTBP1 at the center of a network controlling EMT, DNA-damage responses and cell-death programs in breast cancer.

Recent work has begun to map how CTBP1 activity in breast and other cancers is tuned by post-translational modification. Sahu et al. identified the F-box protein FBXO32 as an E3 ligase that catalyzes K63-linked ubiquitination of CTBP1; this modification is essential for CTBP1’s nuclear retention and its recruitment to promoters of EMT and microenvironmental genes (72). FBXO32 depletion in MDA-MB-231 xenografts reduces nuclear CTBP1, impairs EMT gene induction, and markedly suppresses primary tumor growth and lung metastasis (72). More recently, Lim et al. reported that CTBP1 is modified by the ubiquitin-like protein ISG15, and that ISGylation alters CTBP1’s transcriptional corepressor activity, implicating interferon/innate-immunity pathways in CTBP1 regulation (73).

In addition to CTBP1 protein itself*, CTBP1-AS* and *CTBP1-DT* have been reported to be overexpressed in breast cancer tissues and cell lines, where their silencing suppresses proliferation, migration, and invasion (74, 75). Mechanistically, these antisense transcripts primarily function as competing endogenous RNAs, sequestering tumor-suppressive microRNAs (including miR-940 and miR-381-3p) and thereby sustaining pro-oncogenic transcriptional programs (75). *CTBP1-DT1* (*CTBP1-AS1* in original manuscript) has likewise been shown to promote breast cancer cell proliferation and invasion, consistent with a ceRNA-mediated oncogenic function (76), elevated *CTBP1-DT (CTBP1-AS2* in original manuscript*)* expression correlates with adverse clinicopathological features and poorer prognosis (75), while *CTBP1-AS* has been implicated in the aggressiveness of triple-negative breast cancer (74).

Overall, evidence in breast carcinoma suggests CTBP1 as a central redox- and metabolism-responsive transcriptional corepressor that links cellular metabolic state to epithelial plasticity, stem-like behavior, metastatic spread, and resistance to therapy. Its activity is reinforced by metabolic syndrome, hypoxia, and post-translational regulation, and sustained through both protein-level mechanisms and noncoding RNA networks.

### 5.2 CTBP1 in prostate cancer

In prostate cancer, CTBP1 functions as a metabolically tuned transcriptional oncogene that integrates chromatin repression, metabolic cues, and noncoding RNA regulation. CTBP1 is markedly upregulated and often mislocalized to the cytoplasm in aggressive and metastatic prostate tumors. Functional studies in DU145, PC3 and LNCaP cells show that *CTBP1* KD reduces proliferation, invasion and metastasis, and sensitizes cells to radiation, while xenograft and metastasis models confirm that CTBP1 is required for efficient tumor growth *in vivo* (53). At the transcriptional level, CTBP1 represses multiple tumor suppressors and adhesion genes, including *CDH1* and *CLCA2*, through complexes containing HDACs, ZEB1 and EP300; in metabolic-syndrome-like settings these CTBP1-dependent networks promote EMT, loss of adhesion and increased migration/invasion (54, 77). In mouse models combining high-fat diet and prostate cancer, CTBP1 depletion markedly impairs tumor growth and alters tumor miRNA/mRNA signatures linked to EMT and metabolism, underscoring CTBP1 as a node connecting systemic metabolic syndrome with prostate tumor progression (54, 78).

More recently, post-translational control of CTBP1 has emerged as an additional oncogenic layer in prostate cancer. Normally, CTBP1 binds the transcription factor SP1 at the *CDH1* promoter and represses *CDH1* expression. However, CTBP1 is heavily lysine-succinylated in tumors, and the lysine acetyltransferase KAT2A promotes succinylation of CTBP1 at K46 and K280. This succinylation modulates CTBP1’s transcriptional repressor activity on *CDH1* and enhances prostate cancer cell viability, migration, invasion and glycolysis, while KAT2A KD or mutation of these sites attenuates CTBP1-driven tumor growth *in vivo* (79). These data suggest that CTBP1 may function as a succinylation-sensitive effector within metabolic rewiring programs in prostate cancer.

The androgen-responsive lncRNA *CTBP1-AS*, transcribed antisense to CTBP1, is upregulated in prostate tumors and promotes both hormone-dependent and castration-resistant growth by recruiting the PSF/HDAC complex to repress CTBP1 and additional tumor-suppressor genes, thereby facilitating cell-cycle progression (19). More recently, a second axis was described in which *CTBP1-AS* directly binds TP63 and blocks TP63-mediated activation of the tumor suppressor S100A14, enhancing proliferation, migration, invasion and tumorigenicity in xenograft models. Together, these studies depict CTBP1 in prostate cancer as a metabolically tuned transcriptional corepressor, whose activity is reinforced by succinylation and oncogenic lncRNAs at its own locus, converging on loss of epithelial adhesion, EMT, altered metabolism and metastatic progression (21).

Overall, evidence in prostate cancer identifies CTBP1 as a metabolically tuned transcriptional oncogene that integrates chromatin repression, metabolic rewiring, post-translational control, as well as locus-associated noncoding RNA regulation to promote epithelial plasticity, invasive growth, metastatic progression, and therapy resistance, particularly in metabolically stressed and advanced disease states.

### 5.3 CTBP1 in colorectal cancer

In colorectal cancer, CTBP1 engages early in tumorigenesis and then reinforced by defects in ubiquitin and retinoid signaling. In APC-mutant mouse intestine, and in adenomas from FAP patients, CTBP1 is strongly elevated compared with normal mucosa. APC promotes proteasome-dependent degradation of CTBP1; when APC is lost, CTBP1 accumulates and represses intestinal retinol dehydrogenases (RDHs) required for retinoic-acid (RA) production and epithelial differentiation (80, 81). In complementary models, APC loss produces a two-step adenoma progression phenotype in which CTBP1-mediated repression of RDH/RA signaling contributes to stem-cell overpopulation and incomplete differentiation in colon crypts (49, 82).

In late-stage colorectal cancer, CTBP1 act in the RA-pathway, and control apoptosis. Human colorectal cancer tissues and HT29/SW480 cells show elevated CTBP1 and CYP26A1 together with ALDH1 and RA receptors, whereas these retinoid-pathway components are nearly absent in normal epithelium (83). Functionally, All-trans-retinoic-acid (ATRA) treatment reduces ALDH^+^ colon cancer stem cells, sphere formation and drives differentiation, consistent with CTBP1 acting as a corepressor within an oncogenic RA network that can be therapeutically rebalanced. In parallel, a ubiquitin-proteasome study showed that the E3 ligase HERC5 (via CBP as adaptor) ubiquitinates CTBP1 in non-malignant colon cells. in colorectal carcinoma, HERC5 is downregulated, CTBP1 ubiquitination falls leading to CTBP1 accumulation. Accumulated CTBP1 assembles with HDAC1 and C-MYC into a repressor complex leading a suppression on pro-apoptotic genes *BAX*, *BIK* and *PUMA* (84). Restoring HERC5, knocking down *CTBP1*, or inhibiting CTBP1 with MTOB or NSC95397 derepresses these genes, increases caspase activation, and markedly reduces CRC xenograft growth, especially when combined with oxaliplatin or capecitabine (84). Together, these data supports a model in which CTBP1 functions as a nodal NADH-sensitive corepressor in CRC linking APC mutation, RA metabolism and C-MYC/HDAC1-dependent apoptosis evasion.

The anti-sense-direction transcript *CTBP1-DT (CTBP1-AS2* in original manuscript) is significantly upregulated in colorectal cancer tissues and patient serum; its KD reduces colorectal cancer cell viability, migration and invasion by releasing miR-30a-5p, and serum *CTBP1-DT* (alone or combined with miR-30a-5p) shows good diagnostic performance for distinguishing colorectal cancer from healthy controls (85). The antisense lncRNA *CTBP1-DT* is likewise overexpressed in colorectal cancer, where it promotes proliferation, invasion and xenograft growth by sponging miR-93-5p and sustaining TGF-β/SMAD2/3 signaling (20). Thus, CTBP1 and its locus-encoded lncRNAs together form a hub that integrates APC/Wnt-RA balance, apoptosis control and noncoding RNA circuits in colorectal cancer.

Overall, colorectal cancer studies place CTBP1 as an early and persistent oncogenic corepressor that links intestinal differentiation failure to tumor progression, with its activity reinforced by disrupted protein turnover, altered retinoid signaling, evasion of apoptosis, and locus-associated noncoding RNA circuits, thereby sustaining stem-like states, metabolic adaptation, and therapeutic vulnerability across disease stages.

### 5.4 CTBP1 in lung cancer

In lung cancer, *in vivo* and *ex vivo* data support an oncogenic role for CTBP1 in tumor progression, motility, and microenvironment remodeling. However, the number of studies remains limited. In lung adenocarcinoma cohorts, CTBP1 is elevated in tumors with lymph-node metastasis and associates with adverse clinicopathologic features and shorter overall survival (49). Functionally, shRNA-mediated *CTBP1* depletion in A549 and H1299 cells was reported to reduce proliferation, migration, and invasion as well as increase apoptosis (49). Moreover, *in vivo* patient-derived tumor xenografts further support a growth-promoting role for CTBP1 (49).

A key downstream axis described in nonsmall cell lung carcinoma (NSCLC) involves NF-κB-dependent induction of CCL2, which drives tumor-associated macrophage recruitment and polarization. In A549 and H1299 NSCLC models, *CTBP1* overexpression increases p65 (RelA) activation (including S536 phosphorylation) and nuclear translocation, which is required for CCL2 induction (49). Conditioned media from CTBP1-high NSCLC cells enhances macrophage chemotaxis and promotes a tumor-associated macrophage (TAM)-like state (e.g., increased CD163 and TAM cytokines such as IL-10, CCL17, CCL22), and this effect is attenuated by CCR2 antagonism (sc-202525). In syngeneic mouse experiments, CCR2 blockade (sc-202525) or macrophage depletion (clodronate liposomes) suppresses CTBP1-driven tumor growth and reduces TAM infiltration, positioning CTBP1 as an upstream driver of a CCL2/CCR2-dependent, macrophage-supported NSCLC program (49).

Beyond inflammatory wiring, CTBP1’s canonical corepressor function also maps onto lung carcinoma readouts that are directly relevant to EMT and therapy response. In H1299 lung carcinoma cells, cell-penetrating PLDLS-motif peptides (CPP-E1A-based) disrupt CTBP1 interactions with transcriptional partners (shown for CTBP1-ZEB1) and de-repress epithelial/apoptosis/repair-linked transcripts, including *CDH1*, *BAX*, and *BRCA1*, with concomitant effects on *CDH1* promoter activity (86). This provides a direct experimental route linking CTBP1 partner-binding to repression of EMT- and DNA-damage-relevant genes in a lung cancer context.

Moreover, NSCLC studies also implicate *CTBP1-DT* (*CTBP1-AS2* in original manuscript). In an experimental NSCLC report with luciferase/perturbation-rescue, *CTBP1-DT* was shown to promote malignant phenotypes by acting as a ceRNA, sponging miR-623 to increase MMP3, thereby supporting proliferation/cell-cycle progression and EMT-associated behavior (87).

Overall*, in vivo* and *ex vivo* evidence support an oncogenic role for CTBP1 in lung cancer, with elevated expression associated with tumor progression, metastatic features, and poor clinical outcome, although the number of available studies remains limited. Functional perturbation of CTBP1 consistently suppresses malignant phenotypes in cellular and xenograft models, and emerging evidence suggests additional reinforcement by CTBP1-associated noncoding RNAs.

### 5.5 CTBP1 in glioma

CTBP1 has not been as extensively characterized in glioma as in several epithelial cancers, but recent experimental work places CTBP1-linked corepressor machinery at a key metabolic and epigenetic node in glioblastoma. In patient-derived GBM models and established glioma lines, CTBPs cooperate with the histone demethylase LSD1 in transcriptional control of *SREBP*, fueling *de novo* lipogenesis (88). Mechanistically, ZBTB18, a well-known tumor suppressor in GBM, binds CTBPs via a PLDLS motif and functionally reprograms CTBPs/LSD1 output at *SREBP* gene promoters, resulting in repression of SREBF1/2-associated lipid biosynthesis genes, reduced lipid droplet abundance, and decreased incorporation of glucose-derived carbon into fatty acids/phospholipids (88).

Beyond lipid anabolism, CTBP1 is also associated with glioma aggressiveness phenotypes (58). In human glioma cell models, silencing CTBP1 suppresses migration consistent with CTBP1 participating in transcriptional programs that favor motility and invasion (58). In parallel, depletion of CTBPs in glioma cells, linked CTBP activity to DNA stability/repair signaling (MRN complex integrity and ATR/Chk1-CDK2 axis) and implicated hypoxia-associated circuitry (HIF-1α) downstream of this pathway (89), suggesting that CTBP1-containing complexes may intersect both genotoxic stress responses and tumor microenvironmental pressures in brain tumors.

The lncRNA *CTBP1-DT* (*CTBP1-AS2* in original manuscript) has been reported to associate with adverse clinical features in glioma and to experimentally promote glioma cell proliferation and migration through a defined ceRNA mechanism, in which *CTBP1-DT* sequesters miR-370-3p, leading to derepression of Wnt7a and activation of downstream Wnt/β-catenin-dependent EMT programs (20). This antisense-mediated pathway introduces a second, *CTBP1-*adjacent regulatory layer that converges on EMT and invasion-associated signaling in glioma biology.

Overall, although less extensively studied than in epithelial malignancies, CTBP1 in glioma emerges as a context-dependent oncogenic corepressor linked to metabolic adaptation, invasive behavior, and aggressive disease features, with its activity further reinforced by CTBP1-adjacent noncoding RNA regulation.

### 5.6 CTBP1 in leukemia

In leukemia, specifically in EVI1/MECOM-driven acute myeloid leukemia, CTBP1 functions as a key, druggable cofactor for oncogenic transcriptional repression. Mechanistically, CTBP1 is recruited to chromatin to block differentiation and sustain leukemic programs (90). The requirement for CTBP1 in EVI1-linked transformation was demonstrated experimentally in an early hematopoietic model. The leukemia-associated fusion repressor AML1/MDS1/EVI1 physically interacted with CTBP1 *in vivo*, leading to CTBP1-driven transcriptional repression, and disrupting this interaction impaired abnormal growth and differentiation of murine hematopoietic progenitors (90). More recent work has substantially strengthened and modernized this axis by mapping the EVI1 interactome in acute myeloid leukemia and experimentally testing targetability. Pastoors D. et al., identified CTBP1 (and CTBP2) as among the most enriched EVI1-binding partners, showing that binding is mediated by the PLDLS motif (91). They further used a PLDLS-competitor construct which outcompeted EVI1 binding to CTBPs, suppressed acute myeloid leukemia proliferation *in vitro*, and reduced leukemic growth in xenotransplant settings (91). Although CTBP2 was highlighted as indispensable in that model, CTBP1 is a major binding partner in the same complex and is part of the actionable interaction surface. Accordingly, the strongest targetability data in AML should be interpreted as CTBP-family-level evidence rather than CTBP1-specific dependency. Consistent with CTBP1 being a well-characterized EVI1 partner in acute myeloid leukemia, independent experimental work also showed that EVI1-CTBP1 association is dynamic across the cell cycle in EVI1-overexpressing acute myeloid leukemia cells, suggesting regulated assembly/disassembly of the repressor machinery (92).

Beyond direct leukemic transcription-factor partnering, CTBP-containing complexes (not CTBP1 specific) are increasingly implicated in immune-evasion circuitry in acute myeloid leukemia. Using unbiased CRISPR-Cas9 screening and functional validation, Chan et al. identified the CTBP1 complex as a transcriptional repressor of MHC class II pathway genes in acute myeloid leukemia (93). Moreover, targeting those repressive mechanisms restored MHC-II, boosted antigen-dependent CD4^+^ T-cell responses, and provided a rational strategy to enhance graft-versus-leukemia and immunotherapy-relevant immunity. This mechanism places CTBP1 biology not only in cell-intrinsic leukemic maintenance, but also in the interface between acute myeloid leukemia cells and anti-tumor immune recognition.

Moreover, leukemia’s reliance on glycolysis/redox remodeling makes CTBPs’ classic biochemistry particularly relevant. CTBPs act as NAD(H)-sensitive transcriptional regulators, and experimental work has directly linked aerobic glycolysis-dependent changes in the cytosolic NADH/NAD+ ratio to CTBP-mediated control of transcriptional outputs that intersect with TP53 signaling (31). This provides a mechanistic framework for how glycolytic AML states could tune CTBP1 activity to promote survival and stress tolerance (including therapy responses), even when the most detailed pathway mapping is done outside acute myeloid leukemia.

Although many leukemia subtypes are driven by noncoding RNA programs, *CTBP1-AS* or *CTBP1-DT* has not, to our knowledge, been firmly established as a leukemia driver.

### 5.7 CTBP1 in head and neck squamous cell carcinoma

HNSCC develops within a metabolically harsh, often hypoxic mucosal environment shaped by tobacco/alcohol exposure and (in a subset) HPV-driven oncogenesis (94). These conditions commonly push tumor and premalignant epithelia toward elevated glycolysis and a reduced intracellular redox state (high NADH) (95), precisely the biochemical setting that favors CTBP1 dimerization and stable assembly of repression complexes. In this context, CTBP1 is well positioned to act as a metabolic gatekeeper that translates redox signals into durable transcriptional silencing of tumor-suppressive programs in HNSCC.

*BRCA1* downregulation is detectable early during HNSCC development, a known predictive biomarker of HNSCC (96). Deng Et. al. showed that CTBP1 represses *BRCA1* transcription by binding the *BRCA1* promoter at an E2F4 site, in a redox-sensitive manner (30). In addition, they showed that increasing NADH levels under hypoxic conditions, leads to CTBP1 recruitment to the *BRCA1* promoter (30). The same study also provides clinically relevant histopathology supporting CTBP1 as an early biomarker-like event in squamous carcinogenesis. Using a human HNSCC tissue array, they observed that nuclear CTBP1 staining accumulates already in hyperplastic lesions and HNSCC, and that this nuclear CTBP1 enrichment correlates with *BRCA1* downregulation across matched sections (30). Together, the promoter-level mechanism and tissue correlation argue that CTBP1 is not merely present in HNSCC; it is plausibly functionally engaged early, in a way that fits the metabolic physiology of dysplastic head and neck epithelium.

The Cancer Genome Atlas (TCGA)-based analysis constructing a ferroptosis-related lncRNA prognostic signature for HNSCC identified *CTBP1-DT* among nine key prognostic ferroptosis-related lncRNAs. *CTBP1-DT* behaved as a favorable prognostic factor (97). However, the mechanistic target of *CTBP1-DT* is not yet characterized in HNSCC.

Up to now, BRCA1-mediated DNA repair is the only CTBP1-dependent pathway that has been mechanistically defined in HNSCC. Given that CTBP1 is tightly regulated by cellular metabolism and that HNSCC is among the most hypoxic and metabolically stressed solid tumors, the broader roles of CTBP1 in head and neck tumor biology urgently warrant systematic investigation.

Overall, available evidence supports CTBP1 as an early, metabolism-linked regulatory factor in HNSCC, acting within the hypoxic and redox-stressed mucosal environment characteristic of HNSCC. Its early nuclear accumulation, association with clinically relevant molecular alterations, and linkage to locus-associated noncoding RNAs suggest that CTBP1 may contribute to tumor initiation and progression, while underscoring the need for broader and more systematic investigation of its roles in head and neck cancer.

### 5.8 CTBP1 in ovarian cancer

In ovarian cancer, CTBP family proteins are increasingly appreciated as therapy-response gatekeepers that couple metabolic/redox state to apoptosis competence and DNA-damage handling. Importantly, the available mechanistic studies in this context have not distinguished CTBP1 from CTBP2, and therefore, the evidence should be interpreted as CTBP-family rather than CTBP1-specific. A central experimental theme is that CTBPs’ activity can determine whether ovarian cancer cells engage extrinsic apoptosis (death receptor signaling) versus survive under chemotherapy stress. In ovarian cancer models, CTBPs repress TRAIL death receptors DR4 (*TNFRSF10A*) and DR5 (*TNFRSF10B*), thereby blunting death-receptor-mediated apoptosis (98). Conversely, CTBPs’ inhibition or depletion derepresses these receptors and promotes apoptotic responses, suggesting that CTBPs contribute to apoptosis resistance (98). Moreover, due to the metabolic control of CTBPs oligomerization and its impact on platinum sensitivity, CTBPs dimerization status has been proposed as a biomarker for platinum sensitivity in ovarian cancer (14).

Interestingly, in ovarian cancer, *CTBP1-DT* can be translated (via cap-independent/IRES-dependent mechanisms after DNA damage) into the microprotein DDUP, which sustains DDR signaling and promotes dual repair programs (99). Building on this, in ovarian cancer models, DDUP is strongly induced in cisplatin-resistant cells and is inversely associated with cisplatin response, with mechanistic evidence linking DDUP to persistence of DNA damage repair signaling, thereby promoting platinum resistance (100). Another report described *CTBP1-DT* as functionally relevant through a miR-216a/PTEN axis, where *CTBP1-DT* overexpression suppresses oncogenic phenotypes (101). These findings highlight the context-dependent nature of the *CTBP1* locus. *CTBP1* lncRNA family transcripts can act as oncogenic drivers in some settings and exert tumor-suppressive functions in others, underscoring that *CTBP1*-locus noncoding outputs can have divergent effects depending on cellular lineage and pathway wiring.

Overall, ovarian cancer illustrates how CTBP-family activity may function as a metabolism- and redox-sensitive gatekeeper of therapy response, particularly by influencing apoptotic competence and DNA-damage tolerance. However, it is important to note that direct, CTBP1-specific mechanistic studies in ovarian cancer remain relatively limited, with much of the current evidence deriving from broader CTBP-family analyses or from regulatory outputs encoded at the CTBP1 locus. Available data nonetheless suggest that metabolic control of CTBP activity, together with locus-derived noncoding transcripts, can critically shape platinum responsiveness and stress adaptation in ovarian cancer. This context dependence highlights both the therapeutic potential of targeting CTBP-linked pathways and the need for more focused investigations to disentangle CTBP1-specific functions in this disease.

### 5.9 CTBP1 in esophageal squamous cell carcinoma

In Esophageal Squamous Cell Carcinoma (ESCC), currently available data on CTBP1 are relatively recent, yet they provide direct experimental evidence for a causal role in both chemoresistance and metastatic behavior. A recent CRISPR/Cas9 study knocked out *CTBP1* in paclitaxel-resistant ESCC models (TE-1/PTX and KYSE-50/PTX) and reported that *CTBP1* loss suppresses viability and clonogenicity, delays wound closure, reduces migration/invasion, and sensitizes resistant cells to paclitaxel (102). In the same context, CTBP1 deficiency also enhanced paclitaxel-induced apoptosis, consistent with CTBP1 acting as an upstream transcriptional repressor of epithelial, apoptotic, and differentiation-linked genes in squamous cells (102). These results align well with CTBP1’s classical function as a transcriptional corepressor of epithelial/apoptosis/differentiation programs, and they position CTBP1 as a plausible vulnerability in the neoadjuvant paclitaxel setting.

Interestingly, ESCC currently has stronger evidence for noncoding RNAs that regulate CTBP1 protein itself than *CTBP1*-locus lncRNAs acting as primary drivers. Circular RNA circIMMP2L is a key example of this regulation. circIMMP2L is reported to promote ESCC progression by increasing CTBP1 nuclear retention through HDAC1-mediated deacetylation, leading to increase CTBP1-mediated epigenetic silencing (103). Functionally, the circIMMP2L-HDAC1-CTBP1 axis is associated with increased invasion, lymph node metastasis, and poorer clinical outcome (103), suggesting that in ESCC a noncoding RNA-driven locking of CTBP1 into its nuclear, repressive state can substitute for simple overexpression to achieve an aggressive transcriptional program.

Overall, despite being a relatively recent area of investigation, ESCC provides some of the most direct causal evidence for CTBP1 as a driver of chemoresistance and metastatic behavior. Genetic ablation studies demonstrate dependency in experimental models in maintaining drug-resistant and invasive phenotypes, while emerging noncoding RNA-based mechanisms highlight how CTBP1 activity can be reinforced through altered nuclear retention rather than simple overexpression. Together, these findings suggest CTBP1 as a functionally engaged and potentially targetable vulnerability in aggressive, treatment-refractory ESCC, while underscoring the need for broader validation across patient cohorts.

### 5.10 CTBP1 in osteosarcoma

CTBP1 operates as a transcriptional scaffold consolidating multiple survival signals in osteosarcoma. Early mechanistic studies identified a nuclear complex in which CTBP1 interacts with p300 and FOXO3a to transcriptionally repress pro-apoptotic genes such as *BAX* and *BIM* (45). This axis dampens programmed cell death and promotes osteosarcoma cell survival under stress. Complementing this, CTBP1 is also shown to complex with HDAC1/2 and the interferon regulatory factor IRF1 leading to repression of interferon-stimulated genes, extending CTBP1’s repressor activity into immune-linked transcriptional programs. This IFN axis shed lights on CTBP1’s role in immune-adaptive phenotypes and proliferative advantage in osteosarcoma cells beyond its canonical roles in EMT and stemness (65). Moreover, CTBP1 is involved in the FOXM1 complex in osteosarcoma cancer stem-like cells. This interaction has been functionally linked to the transcriptional activation of *MDR1* and additional chemoresistance-associated genes, stabilizing survival programs enriched in therapy-resistant subpopulations (104). Disruption of the CTBP1-FOXM1 axis leads to a reduction in *MDR1* expression and impairs the viability of aggressive cell states (104). More recently, CTBP1-containing complexes have been linked to DDR modulation and therapy resistance. A study centered on the DNA repair protein CtIP revealed that CtIP recruits CTBP1/2 and HDAC1 to repress AP-1 transcriptional activity (105). This repression facilitates osteosarcoma cell survival in the face of DNA damage and chemotherapy, reinforcing a broader pattern in which CTBP1-based complexes buffer cellular stress by throttling transcriptional output from damage-sensing pathways (105).

Although lncRNAs transcribed from the CTBP1 locus have been shown to play critical regulatory roles in multiple cancer types, their involvement in osteosarcoma has not yet been experimentally investigated.

Overall, available evidence positions CTBP1 as a central survival-supporting regulator in osteosarcoma, consolidating stress tolerance, immune adaptation, and therapy resistance across aggressive tumor cell states. While mechanistic studies highlight a clear requirement for CTBP1-containing complexes in sustaining viability under apoptotic, immune, and genotoxic pressure, the contribution of CTBP1-locus noncoding RNAs in osteosarcoma remains unexplored, marking an important gap for future investigation.

### 5.11 CTBP1 in melanoma

Melanoma presents a nuanced, somewhat paradoxical CTBP1 story that appears to reflect melanoma’s well-known phenotype switching (proliferative vs invasive states). On one hand, CTBP1 is clearly expressed in melanoma and can act as an oncogenic corepressor of canonical tumor suppressor programs (106). A key study showed CTBP1 represses *p16INK4a*, and *BRCA1* as well as an inverse relationship between *CTBP1* overexpression and *BRCA1* loss in malignant melanoma tissues was reported (52), connecting CTBP1 to both proliferation control and compromised DNA repair capacity. On the other hand, an earlier melanoma-focused report observed that loss of *CTBP1* correlated with increased migratory/invasive potential, suggesting CTBP1 can behave as a molecular brake on invasion in certain melanoma contexts, including upregulation of melanoma inhibitory activity protein (MIA) (107, 108). This paradoxical situation suggests CTBP1’s role depends on which transcriptional programs dominate the melanoma state (e.g., repressing differentiation and checkpoint genes to favor growth, while also potentially repressing invasion-linked secreted factors such as MIA in specific settings). This is in line with melanoma’s plasticity and indicates that CTBP1 targeting could shift state behavior, not simply, reduce all malignancy outputs. These findings suggest that CTBP1 inhibition may not uniformly suppress melanoma malignancy, but instead shift tumor cells between proliferative and invasive states. Therapeutic targeting may therefore require combination strategies to prevent state-transition-mediated escape.

For *CTBP1* locus lncRNAs, melanoma remains comparatively underdeveloped mechanistically. Current melanoma literature has emphasized CTBP1 protein and its repression targets, while *CTBP1* locus noncoding transcripts have not yet been established as central melanoma drivers.

Finally, melanoma is one of the most compelling translation-to-therapy contexts for CTBP1 pharmacology. A very recent study in 2024 reported a potent and selective CTBP1 inhibitor (N-(3,4-dichlorophenyl)-4-[((4-nitrophenyl)carbamoyl)amino]benzenesulfonamide) with activity in cellular systems and melanoma animal models, showing that CTBP1 is not only mechanistically interesting but also druggable *in vivo* (109). Upon binding to CTBP1, inhibitor: (i) induces cell cycle arrest by regulating genes like *CDKN2A*, *CDKN1A*, *p14ARF*, and *CCND1*; (ii) promotes apoptosis through modulation of p53, PTEN, BRCA1, and BRIP1; (iii) suppresses mesenchymal traits, shifting gene expression toward an epithelial profile and reducing invasiveness; and (iv) inhibits primary tumor growth *in vivo*.

Overall, melanoma highlights a context-dependent and state-specific role for CTBP1, consistent with the pronounced phenotypic plasticity of this disease. While CTBP1 can function as an oncogenic corepressor supporting proliferative growth and survival programs, evidence also indicates that its loss may favor invasive behavior in certain settings, underscoring a paradox that reflects melanoma state switching rather than a simple oncogene-tumor suppressor dichotomy. A key limitation is that *CTBP1*-locus noncoding RNAs remain poorly defined in melanoma, leaving open whether *CTBP1* locus-encoded transcripts also modulate melanoma or CTBP1 independently shapes phenotype switching. Notably, emerging pharmacological work demonstrating *in vivo* CTBP1 druggability strengthens the translational relevance of this axis, but also emphasizes the need to understand how CTBP1 targeting might shift tumor state, not merely suppress growth.

### 5.12 CTBP1 in pancreatic ductal adenocarcinoma

In pancreatic ductal adenocarcinoma (PDAC), CTBPs act as a metabolically regulated transcriptional corepressor leading to tumor initiation, invasive progression, and chemoresistance. Both CTBP1 and its paralog CTBP2 are found to be highly expressed in human PDAC tumors (59). Functional studies suggested CTBP1 as a limiting factor for PDAC growth. Partial genetic ablation of CTBPs (CTBP2 haploinsufficiency, with concurrent reduction of CTBP1) in a mutant KRAS-driven PDAC model significantly prolonged survival and abrogated peritoneal metastasis (59). This was accompanied by a dramatic downregulation of *CMYC*, consistent with CTBP1/2 supporting an oncogenic stem-like state. At the mechanistic level, CTBP1’s well-known NADH-dependent dimerization underlies its activity as a sensor of the high-glycolytic/redox environment in PDAC. However, the study includes both CTBP1 and CTBP2, causing a problem to distinguish CTBP1’s role in PDAC. Importantly, the available *in vivo* and mechanistic data do not disentangle CTBP1 from CTBP2, and therefore, current evidence should be interpreted as CTBP-family rather than CTBP1-specific. To date, no CTBP1-specific study in PDAC has been established.

On the other hand, the roles of CTBP1-associated antisense lncRNAs in PDAC are well studied. Notably, *CTBP1-DT* (*CTBP1-AS2* in original manuscript) is markedly overexpressed in pancreatic carcinoma tissues and correlates with advanced stage and lymph-node metastasis (110). Silencing *CTBP1-DT* in PDAC cell models inhibits proliferation, migration, and invasion, while promoting apoptosis, indicating its oncogenic function (110). Mechanistically, *CTBP1-DT* acts as a competing endogenous RNA, sponging miR-141-3p, leading to the release of post-transcriptional repression of the deubiquitinase USP22 (110). The resulting upregulation of USP22 is thought to stabilize pro-EMT and anti-apoptotic programs.

Overall, PDAC positions CTBPs rather than CTBP1 alone as a metabolically tuned oncogenic axis that supports tumor growth, invasion, and metastatic competence in a highly glycolytic, redox-stressed environment. While genetic evidence from KRAS-driven models implicates CTBPs as limiting factors for PDAC progression, direct CTBP1-specific mechanistic studies remain lacking, making it difficult to disentangle CTBP1 from CTBP2 contributions. In contrast, *CTBP1*-locus noncoding RNAs, particularly *CTBP1-DT*, are well characterized and clearly oncogenic in PDAC, highlighting a setting in which locus-encoded regulatory outputs are better defined than CTBP1 protein-specific functions and underscoring a key gap for future investigation.

### 5.13 CTBP1 in hepatocellular carcinoma

In Hepatocellular Carcinoma (HCC), CTBP1 has been shown to contribute to EMT and to support tumor cell survival under stress. Clinically, CTBP1 is significantly upregulated in HCC tissues and shows an inverse correlation with E-cadherin protein levels (63). In other cancer models, CTBP1 is known to function as a corepressor for *CDH1* transcription by partnering with EMT transcription factors (such as ZEB1/2) at the *CDH1* promoter (111). In line with that, CTBP1-high HCC cells exhibit loss of cell-to-cell adhesion and enhanced invasiveness. KD of *CTBP1* in HCC cell lines (such as HepG2) leads to upregulation of *CDH1*, a marked reduction in migratory and invasive ability, as well as an increase in apoptosis, without causing cell-cycle arrest (63). These findings suggest that CTBP1 contributes to maintenance of the mesenchymal, anti-apoptotic state in HCC cells. CTBP1 has also been identified as a redox-sensitive, oxygen sensor, mediating hypoxia-driven EMT (sarcomatoid transformation) in HCC (112). Under low-oxygen (hypoxia), high NADH conditions, CTBP1 is recruited to repress epithelial genes and induce a spindle-cell, motile phenotype (112). This transition is abrogated by CTBP1 inhibition (112). This phenomenon underscores that CTBP1 links the HCC microenvironment (e.g. hypoxia and metabolic stress) to aggressive transcriptional reprogramming.

Beyond cell adhesion programs, CTBP1 in HCC directly interfaces with metabolic and DNA damage response pathways to confer therapy resistance. A recent study showed that CTBP1 forms a complex with HDAC1/2 to repress *MAT1A* (methionine adenosyltransferase-1A) which is crucial for generating S-adenosylmethionine in normal liver (55, 113). CTBP1-driven *MAT1A* silencing has multifaceted pro-tumor consequences. Loss of *MAT1A* in HCC is known to suppress ferroptosis (iron-dependent cell death) and enable immune evasion (55, 113). Accordingly, CTBP1-overexpressing HCC cells display reduced ferroptotic damage and diminished CD8^+^ T cell cytotoxicity in the tumor microenvironment (55). Rescue of *MAT1A*, or experimental disruption of the CTBP1/HDAC complex, abolishes these effects and restores immune- and ferroptosis-associated sensitivity (55). This suggests that CTBP1 may act as an antagonist of both tumor immunity and ferroptosis, two emerging axes of therapy response in liver cancer.

On the noncoding RNA, *CTBP1-DT* (*CTBP1-AS2* in original manuscript) is highly upregulated in HCC and was found to be transcriptionally activated by SP1 (55). *CTBP1-DT* acts as a ceRNA to sponge tumor-suppressor microRNAs, leading enhancement of proliferative and survival signals. For instance, *CTBP1-DT* can sequester miR-623, leading to upregulation of *CCND1* (Cyclin D1) and accelerated cell cycle progression in HCC cells (55). Similarly, *CTBP1-DT* binding to miR-195-5p (another downregulated miRNA in HCC) derepresses *CEP55*, a centrosomal protein involved in cytokinesis and implicated in tumor growth and chromosomal instability (55). Functionally, *CTBP1-DT* KD suppresses HCC cell proliferation, migration, and invasion, in some studies also reducing EMT markers and enhancing chemosensitivity (55). Taken together, CTBP1 and its antisense lncRNA form a malignant feedback loop in HCC. CTBP1 fosters an EMT, apoptosis-resistant, immune-evading tumor state, and *CTBP1-DT* further insulates and amplifies this state by blocking miRNA brakes on key oncogenic genes.

Overall, hepatocellular carcinoma suggests CTBP1 as a central driver of aggressive tumor behavior, linking elevated expression to loss of epithelial identity, enhanced invasion, stress tolerance, and poor therapeutic responsiveness in a hypoxic and metabolically challenged liver environment. Clinical and experimental evidence supports a role for CTBP1 in sustaining survival, immune evasion, and resistance to emerging cell-death pathways, while CTBP1-locus noncoding RNA, *CTBP1-DT*, further reinforces malignant phenotypes. Together, these findings position CTBP1 as a key integrator of microenvironmental stress and oncogenic adaptation in HCC, with clear relevance for disease progression and treatment response.

### 5.14 CTBP1 in gastric cancer

In gastric cancer, CTBP1 operates as a transcriptional corepressor associated with metastasis and drug resistance. Elevated CTBP1 expression is observed in gastric cancer tissues and cell lines, correlating with more aggressive phenotypes (114, 115). CTBP1 is known to promote EMT and invasion by repressing epithelial genes and activating pro-migratory pathways (115). In a clinical study it was found that CTBP1 is upregulated in ~70% of primary gastric cancer tumors and that high CTBP1 levels are associated with increased tumor size, lymphovascular invasion, and distant metastasis (114). Mechanistically, CTBP1 was shown to modulate the JAK/STAT signaling axis in gastric carcinoma. Overexpression of CTBP1 hyperactivated JAK1/Stat3 signaling, while CRISPR/Cas9-mediated KO of *CTBP1* significantly reduced Stat3 phosphorylation, resulting in impaired gastric cancer cell migration, invasion, and colony formation (114). *CTBP1* loss *in vivo* notably constrained metastasis in gastric cancer models, suggesting that CTBP1’s pro-EMT transcriptional programs feed into pathways (like JAK1/STAT3) that orchestrate tumor cell motility and metastasis (114). Thus, CTBP1 appears to function at an interface between intrinsic transcriptional repression and extrinsic signaling pathways in gastric cancer. These data were further validated *in vitro*, where miRNA-mediated loss of *CTBP1* leads to decreased metastasis and proliferation (115).

CTBP1 also plays significant roles in gastric cancer chemoresistance, particularly resistance to platinum-based therapy. In cisplatin-resistant gastric cancer cell lines, CTBP1 expression is found to be markedly higher than in parental cells (56). *CTBP1* depletion in these drug-resistant gastric cancer cells (AGS/DDP, HGC-27/DDP) causes a significant resensitization to cisplatin. Cell proliferation declines and apoptosis increases upon *CTBP1* KD (56). A key effector of this resistance phenotype is the DNA repair protein RAD51. CTBP1 was shown to transcriptionally upregulate RAD51 in cisplatin-resistant cells, thereby enhancing the homologous recombination repair capacity (56). Overexpression of RAD51 can rescue the viability of CTBP1-depleted GC cells under cisplatin treatment, confirming that CTBP1’s promotion of DNA-damage repair is a major mechanism of chemoresistance (56). This aligns with CTBP1’s broader role in suppressing apoptotic genes and sustaining survival pathways.

*CTBP1-DT* (*CTBP1-AS2* in original manuscript) is found to facilitate gastric tumor progression. *CTBP1-DT* is significantly overexpressed in gastric cancer tissues, with higher levels strongly associating with advanced TNM stage, larger tumor size, and poorer differentiation (116). Functional assays demonstrate that *CTBP1-DT* promotes gastric cancer cell proliferation and metastasis while inhibiting apoptosis (116). At the molecular level, *CTBP1-DT* operates through a miR-139-3p/MMP11 (matrix metalloproteinase-11) axis. *CTBP1-DT* sponges miR-139-3p, a microRNA that normally suppresses MMP11 and other pro-EMT genes. By sequestering miR-139-3p, *CTBP1-DT* upregulates MMP11, which in turn drives extracellular matrix degradation, invasion, and EMT in gastric cancer (116). Through *CTBP1-DT* the *CTBP1* locus thus reinforces a feed-forward loop in gastric cancer. The corepressor activity of CTBP1 (silencing epithelial and pro-apoptotic genes) is sustained and enhanced by a lncRNA-mediated microRNA sponge that frees key EMT effectors like MMP11. This multilayered regulation contributes to both the metastatic capacity and the therapy resistance (e.g. to cisplatin) of gastric cancer cells.

Overall, gastric cancer studies suggest CTBP1 as a transcriptional driver of tumor aggressiveness, with elevated expression linked to metastatic progression and resistance to platinum-based therapy. Experimental and clinical data support a role for CTBP1 in sustaining invasive and drug-tolerant tumor states, while *CTBP1-DT* further reinforces these malignant phenotypes. Together, these findings define CTBP1 as a key integrator of transcriptional repression and therapy resistance in gastric cancer, with relevance for both disease progression and treatment failure.

### 5.15 CTBP1 in cervical cancer

Cervical cancer, largely driven by HPV oncoproteins, has only recently been examined for CTBP1-related mechanisms. While direct studies of CTBP1 protein in cervical carcinoma are still limited, the *CTBP1* locus encodes an antisense lncRNA that clearly contributes to cervical cancer aggressiveness. *CTBP1-DT* (*CTBP1-AS2* in original manuscript) is significantly overexpressed in cervical cancer tissues and cell lines (117). Functionally, *CTBP1-DT* acts as an oncogenic driver in cervical cancer. Its KD suppresses cervical cancer cell proliferation, impedes migration/invasion, and induces robust apoptosis (117). Mechanistically, *CTBP1-DT* in cervical cancer cytoplasm serves as a sponge for miR-3163 (117). By binding and sequestering miR-3163, *CTBP1-DT* prevents this microRNA from downregulating its target, the ZNF217 oncogene (117).

Given the oncogenic role of *CTBP1-DT* in cervical cancer, it is crucial to investigate whether the CTBP1 protein itself contributes functionally to cervical carcinogenesis, particularly in the context of HPV-driven transcriptional reprogramming. While lncRNA-mediated effects are increasingly clear, the corepressor functions of CTBP1, such as repression of tumor suppressors, modulation of redox-sensitive transcription, remain unexplored in cervical models. Furthermore, the overlap between CTBP1’s established roles in EMT, apoptosis suppression, and DNA repair in other cancers suggests that it may similarly facilitate tumor progression, immune evasion, or therapy resistance in cervical cancer. Defining whether CTBP1 protein and *CTBP1-DT* function independently, cooperatively, or within a feedback loop in cervical carcinoma could uncover novel therapeutic vulnerabilities.

Overall, cervical cancer remains poorly characterized with respect to CTBP1 protein function, but emerging evidence supports *CTBP1*-locus noncoding RNA activity as a clear driver of tumor aggressiveness. While *CTBP1-DT* shows a well-defined oncogenic role, the contribution of CTBP1 itself in cervical carcinogenesis remains unexplored, representing an important gap with potential therapeutic relevance.

### 5.16 CTBP1 in clear cell renal cell carcinoma

In clear cell renal cell carcinoma (ccRCC), CTBP1 protein has only recently begun to be examined, and its transcriptional corepressor functions have not yet been systematically mapped. Specifically, *LINC01426* was shown to modulate CTBP1 abundance and activity through interaction with IGF2BP1, enabling CTBP1 to participate in transcriptional repression programs that promote ccRCC cell proliferation and invasion (118). However, these findings arise from a lncRNA-centered framework, and CTBP1 has not yet been independently characterized with respect to its endogenous expression patterns, subcellular localization, or genome-wide transcriptional targets in ccRCC cells or tissues. In parallel, CTBP1-locus noncoding RNA activity is more extensively documented.

*CTBP1-DT* has been reported to promote ccRCC cell survival and aggressiveness. A recent experimental study, demonstrated that *CTBP1-DT* is significantly upregulated in ccRCC tumors compared with normal kidney tissue, and its expression correlates with immune-related gene signatures and patient prognosis (119). Functionally, *CTBP1-DT* KD suppressed lipid accumulation, reduced proliferation and migration, and enhanced apoptosis in ccRCC cell lines, implicating *CTBP1-DT* in two hallmarks of ccRCC biology; metabolic rewiring and apoptosis resistance (119). Although paradoxically associated with favorable immune infiltration and survival in some patients, *CTBP1-DT* clearly operates as a tumor cell-intrinsic survival factor, warranting further investigation into its interactions with the CTBP1 protein or its potential role in modulating CTBP1’s activity. The absence of protein-level studies in this context leaves an open question as to whether *CTBP1-DT* exerts regulatory control over CTBP1 or functions independently.

Overall, ccRCC remains a setting in which CTBP1 protein function is essentially unexplored, with no direct evidence yet addressing its expression, localization, or transcriptional activity in renal cancer models. In contrast, *CTBP1-DT* has emerged as functionally relevant, promoting tumor cell survival, metabolic adaptation, and resistance to apoptosis. The coexistence of clear locus-level oncogenic effects and a complete absence of protein-level data highlights a critical gap in the field, leaving unresolved whether *CTBP1-DT* operates in line with CTBP1 activity or operates through independent regulatory circuits in ccRCC.

### 5.17 CTBP1 in cholangiocarcinoma

Cholangiocarcinoma has no experimental evidence directly linking CTBP1 protein to tumor progression, no studies to date have examined the role of CTBP1 protein itself in cholangiocarcinoma biology. Neither CTBP1 expression patterns nor its transcriptional repression complexes have been mechanistically mapped in cholangiocarcinoma models. However, the *CTBP1* locus encodes *CTBP1-DT* (*CTBP1-AS2* in the original manuscript), which has been experimentally validated. Recently it has been shown *CTBP1-DT* is significantly overexpressed in cholangiocarcinoma tumors and is regulated through m^6^A RNA methylation by the nuclear reader protein YTHDC1 (120). This YTHDC1/*CTBP1-DT* interaction enhances CTBP1-DT stability and function. KD of either *CTBP1-DT* or *YTHDC1* in cholangiocarcinoma cells induces cell-cycle arrest, reduces cell proliferation and invasion, and promotes apoptosis, highlighting a functional role for *CTBP1-DT* in sustaining malignancy (120). However, the mechanistic target remains unclear. Thus, while *CTBP1-DT* has established importance in cholangiocarcinoma, the contribution of CTBP1 protein to cholangiocarcinoma progression remains entirely unexplored.

Overall, cholangiocarcinoma remains unexplored with respect to CTBP1 protein function, with no direct experimental data addressing its expression or transcriptional activity. In contrast, *CTBP1-DT* has clear functional relevance, promoting proliferation, invasion, and survival, underscoring a major gap in understanding how, or whether, CTBP1 protein contributes to cholangiocarcinoma progression.

## 6 Therapeutic considerations and limitations

General limitations: Although CTBP1 is increasingly recognized as an oncogenic factor, targeting CTBP1 presents several challenges. General obstacles include the lack of highly specific CTBP1 inhibitors and functional redundancy with its homolog CTBP2. For example, commonly used CTBP1/2 dehydrogenase pocket inhibitors (such as MTOB and 4-Cl-HIPP) have significant off-target effects. A recent study showed these compounds induced broad transcriptional changes even in *CTBP1/2*-KO cells (121). Thus, efficacy data from such inhibitors must be interpreted with caution. Moreover, CTBP2 can compensate for loss of CTBP1 *in vivo* (8), so inhibiting CTBP1 alone may be insufficient unless CTBP2 is co-targeted. CTBP1 is also a metabolic sensor and transcriptional corepressor for numerous genes; systemic CTBP1 inhibition could disrupt normal metabolic and developmental processes (e.g. adipogenesis, differentiation) (122). Delivering CTBP1-targeted therapies is another hurdle. For instance, peptide inhibitors or NADH mimetics would need sufficient bioavailability and tissue penetration (crossing the blood-brain barrier in gliomas). Finally, no established biomarkers exist to identify patients most likely to benefit from CTBP1 inhibition.

Below, we discuss cancer type-specific gaps and limitations in targeting *CTBP1* gene (either the protein or associated noncoding RNAs) in various tumor contexts.

Breast cancer-specific limitations: In breast cancer, CTBP1’s role in metabolic regulation raises concerns that its inhibition could have unintended effects on normal cells. CTBP1 represses sterol regulatory element-binding factor 2 (*SREBF2*), reducing intracellular cholesterol levels and thereby promoting TGF-β signaling and metastasis (67). Inhibiting CTBP1 would derepress *SREBF2*, potentially increasing cholesterol synthesis and altering membrane composition in normal tissues. This is especially relevant in hormone-sensitive breast cancers. CTBP1 activity is tied to estrogen-related metabolism as well. For example, CTBP1 can repress aromatase and other genes in steroidogenic tissues (123). Thus, systemic CTBP1 inhibition might unintentionally increase estrogen production or disrupt adipocyte function. These metabolic and endocrine side effects represent a significant limitation. Furthermore, while *CTBP1* KD in breast cancer cells can impede migration and induce tumor suppressor genes, no study has yet tested a selective CTBP1 inhibitor in breast cancer models. The evidence mostly comes from genetic manipulations. Without such data, it remains unclear how to target CTBP1 safely in breast cancer patients. In summary, CTBP1’s integration in metabolic homeostasis (cholesterol, adipocyte signaling) complicates therapeutic targeting in breast tumors.

Prostate cancer-specific limitations: Prostate tumors often express both *CTBP1* and *CTBP2*, which may substitute for each other. High CTBP1 levels correlate with aggressive disease, and *CTBP1* KD can suppress prostate cancer growth and metastasis in models (53). However, CTBP2 is frequently upregulated in prostate cancer as well (124). This redundancy brings the idea that it might not be sufficient to inhibit CTBP1 alone, CTBP2 could continue driving oncogenic pathways. Another challenge is CTBP1’s context-dependent role in androgen receptor AR signaling. CTBP1 can function as a corepressor for AR-responsive genes. Notably, an androgen-induced lncRNA (*CTBP1-AS*) promotes prostate tumor growth by directly repressing CTBP1, thereby enhancing AR signaling and castration-resistant growth (19, 125). This indicates that in AR-dependent prostate cancer cells, CTBP1 actually constrains certain oncogenic programs. Consequently, broad CTBP1 inhibition could unleash AR-driven transcription in hormone-sensitive cells, potentially accelerating tumor growth in that subset (102). On the other hand, in AR-independent or neuroendocrine prostate cancer, CTBP1’s protumor effects (e.g. repressing pro-apoptotic genes) likely dominate (53). Navigating this dichotomy is a major limitation. CTBP1-targeted therapy might require combination strategies or patient selection based on AR pathway status.

Lastly, as CTBP1 is involved in normal prostate development and differentiation; systemic inhibition might have off-target toxicity in prostate tissue. To date, no clinical or preclinical inhibitor studies have been reported in prostate cancer, so these theoretical issues remain unaddressed.

Colorectal cancer-specific limitations: In colorectal cancer, CTBP1 is a context-dependent transcriptional modulator of the Wnt pathway and cell cycle genes. Preclinical studies show that CTBPs suppression (genetic or via 4-CI-HIPP analogues) can reduce intestinal polyp formation in APC-mutant models (102), supporting CTBP1 as a target in Wnt-driven colorectal cancer. However, CTBP1 also helps maintain normal intestinal stem cell homeostasis. CTBP1 represses multiple tumor suppressors (e.g. *CDH1* and *CDKN2A*). Inhibiting CTBP1 could thus derepress these genes in both cancer cells and normal intestinal epithelium. While re-expression of *CDH1* or *CDKN2A* is desirable in tumors, in normal stem cells it could cause premature differentiation or senescence. This raises the risk of mucosal toxicity or impaired regeneration. Additionally, CTBP1/2 are broadly expressed in gut tissues and influence metabolic gene networks (102, 126, 127). One study noted that partial CTBP inhibition in APC-mutant mice, although reducing polyps, also decreased the normal stem cell pool in the intestines (128). Thus, targeting CTBP1 in colorectal cancer may have a narrow therapeutic window between antitumor efficacy and disruption of gut homeostasis.

Another limitation is the lack of protein-level validation in some contexts. CTBP1 has been studied more in cell lines and mouse models than in human colorectal cancer specimens. Biomarkers to identify colorectal cancer patients who rely on CTBP1 are still needed. No CTBP1 inhibitor has yet advanced to colorectal cancer clinical trials, underscoring the gap between experimental promise and therapeutic reality.

Lung cancer-specific limitations: In NSCLC, a key challenge is that CTBP1 influences the tumor microenvironment, particularly immune cell recruitment. CTBP1 was recently shown to promote TAM infiltration in NSCLC by upregulating CCL2 via NF-κB signaling (49). In theory, CTBP1 inhibition could reduce immunosuppressive TAMs and benefit therapy. However, this role also means CTBP1 intersects with innate immune pathways. CTBP1 is subject to ISGylation (ISG15 modification) in response to interferon, enhancing its corepressor activity on immune-related genes (73). Disrupting CTBP1 might therefore alter cytokine responses or antiviral defenses; an unintended immunomodulation.

From a drug delivery standpoint, lung tumors present unique difficulties. CTBP1 inhibitors would need to penetrate often fibrotic tumor stroma and possibly act on both cancer cells and infiltrating immune cells. Off-target effects on normal lung cells are also a concern. CTBP1 is expressed in some pulmonary cells and may play roles in alveolar cell survival or response to injury. We also note that, to date, no studies have directly targeted CTBP1 protein in lung cancer models. The evidence for CTBP1’s importance comes from correlative expression data and *in vitro* KDs (e.g. higher CTBP1 levels correlate with worse NSCLC survival and KD reduces invasion *in vitro*) (49),. Thus, a limitation in lung cancer is simply proof-of-concept. it remains to be shown whether a CTBP1-targeted agent can be delivered effectively to lung tumors and produce an anticancer immune effect without harming normal lung function.

Glioma-specific limitations: Targeting CTBP1 in gliomas faces the formidable obstacle of the blood-brain barrier (BBB). CTBP1 inhibitors that are large or hydrophilic (such as peptide antagonists or NAD+/NADH analogues) (86) may have poor BBB penetration. Even small molecules would need to achieve sufficient brain concentrations to affect glioma cells diffusely infiltrating brain tissue. Furthermore, CTBP1 has essential roles in neurons. For instance, CTBP1 participates in activity-dependent gene repression in neurons and in pain modulation by silencing opioid receptor gene expression in dorsal root ganglia (129). In the central nervous system, CTBP1 helps regulate synaptic function and plasticity (41). Inhibiting CTBP1 in a patient could potentially de-repress neuronal genes such as the μ-opioid receptor, as seen in neuropathic pain models (129), or broadly affect synapse-associated gene expression. The result could be neurotoxicity or cognitive side effects, an especially important consideration for glioblastoma patients where aggressive treatment can already impair neurologic function. Additionally, CTBP1 is implicated in maintaining neural stem cell quiescence and differentiation; its loss might perturb neurogenesis (130, 131). To date there have been no trials of CTBP1-targeted drugs for glioma, so these theoretical limitations, BBB delivery and on-target neural effects, remain unresolved. Any future CTBP1 inhibitor for brain tumors would likely need structural modifications or delivery mechanisms (e.g. convection-enhanced delivery or BBB disruptors) to reach the tumor, while sparing normal brain regions as much as possible.

Leukemia-specific limitations: No studies have yet directly targeted CTBP1 protein in leukemias, but functional data suggest caution. CTBP1 is expressed in hematopoietic progenitors and helps regulate the balance of differentiation and self-renewal. Complete loss of CTBP1 in mice is viable, but loss of both CTBP1 and CTBP2 is embryonic lethal (8), indicating at least partial redundancy in blood development. In acute leukemia, CTBP1 often operates as a cofactor for oncogenic transcriptional repressors (90). For example, the AML1-ETO fusion protein in acute myeloid leukemia recruits CTBP1 via a PLDLS motif to silence myeloid differentiation genes (90). This suggests that inhibiting CTBP1 could disrupt leukemic repression complexes and promote differentiation, a potential therapeutic angle. However, CTBP1 also influences normal immune cell function. CTBP1’s transcriptional activity is modulated by innate immune signaling; it becomes ISGylated upon interferon stimulation, which enhances its repression of interferon-stimulated genes (73). If a CTBP1 inhibitor were given systemically, it might impair the interferon/ISG15 pathway and thus the patient’s antiviral and antimicrobial immunity. Another practical limitation in leukemia is the protective niche environment. Leukemia stem cells reside in bone marrow niches that are difficult for many drugs to penetrate (132). A CTBP1-targeted agent would need adequate bone marrow exposure and might need to be combined with niche-disrupting strategies. Finally, CTBP1 is involved in DNA damage responses and radio-resistance in cancer cells (53); inhibiting it could sensitize leukemia cells to therapy, but also risk bone marrow toxicity. Without any *in vivo* studies yet, these remain theoretical concerns. Any CTBP1-targeted approach in leukemia must be carefully evaluated for hematopoietic toxicity and immune suppression.

HNSCC-specific limitations: No studies thus far have selectively drugged CTBP1 protein in HNSCC, but recent findings highlight CTBP1-associated long noncoding RNAs in this cancer. Notably, *CTBP1-DT* was identified as a ferroptosis-related lncRNA that may influence HNSCC outcomes (97). *CTBP1-DT* (*CTBP1-AS2* in original manuscript) is significantly upregulated in HNSCC tumors and has been linked to ferroptotic cell death pathways (97). The exact function of *CTBP1-DT* is still under investigation, but it appears to modulate redox balance and iron metabolism in HNSCC cells. This raises a limitation for targeting the CTBP1 axis. if *CTBP1* is inhibited without understanding the role of *CTBP1-DT*, the result could be unpredictable.

Another issue is the delivery of CTBP1 inhibitors in head and neck cancer. These tumors are often localized to mucosal surfaces (oral cavity, pharynx), suggesting that topical or local delivery might maximize drug concentration while minimizing systemic exposure. However, local delivery (e.g. intra-tumoral injection or mouthwash formulations) would need to ensure adequate drug penetration in larger or invasive lesions. There is also the concern of collateral damage to normal mucosal cells and stromal fibroblasts which also express CTBP1 (133, 134). Inhibiting CTBP1 in normal oral epithelium could, for example, impair wound healing or the integrity of the mucosal barrier, leading to ulceration or infection risk. In summary, for HNSCC the limitations include a lack of clarity on CTBP1’s interplay with lncRNAs like *CTBP1-DT*, and practical challenges in safely delivering the therapy to the tumor site.

Osteosarcoma-specific limitations: Osteosarcoma research illustrates both the promise and limitations of CTBP1 targeting. CTBPs are highly expressed in some osteosarcoma cell lines and have been shown to repress key genes (e.g. *CDKN2A*, *BRCA1*) that constrain tumor growth (52). Targeting CTBP1 can restore these tumor suppressors. In fact, one study demonstrated that disrupting the CTBP1-FOXM1 complex (with small-molecule inhibitors) reversed MDR1-mediated drug resistance in osteosarcoma stem-like cells (104). This indicates CTBP1 inhibition might chemosensitize tumors. However, patient tumor data reveal a nuance: CTBP2, not CTBP1, is often the dominantly upregulated isoform in osteosarcoma tissues (135). High CTBP2 levels associate with metastatic progression in OS, whereas CTBP1 levels are less frequently elevated (135). Therefore, a CTBP1-specific drug might yield limited benefit if CTBP2 continues to drive the malignant phenotype. This redundancy is a major limitation in OS. Effective therapy might require dual inhibition of CTBP1 and CTBP2, or a broad disruption of the CtBP family’s binding interface. Another gap is *in vivo* evidence. While CTBP1 inhibitors (like NSM00158) curbed osteosarcoma xenograft growth and overcame cisplatin resistance in mice (104), these compounds target CTBP1’s dehydrogenase pocket non-selectively (they likely target CTBP2 as well). Off-target effects or toxicity were not deeply reported. Osteosarcoma predominantly affects adolescents, so any CTBP1-targeted therapy must be assessed for long-term developmental side effects, especially since CTBP proteins can influence bone growth and repair. In summary, osteosarcoma studies underscore isoform compensation (CTBP2 upregulation) and the need for further validation of CTBP1 inhibitors’ safety.

Melanoma-specific limitations: Melanoma cells exploit CTBP1’s broad transcriptional repression to sustain proliferation. CTBP1 is overexpressed in melanoma and directly represses the tumor suppressors *CDKN2A* and *BRCA1* (52). By silencing *CDKN2A*, CTBP1 helps melanoma cells bypass senescence checkpoints (52). This identifies CTBP1 as a potential target, restoring p16INK4A (*CDKN2A*) and BRCA1 levels via CTBP1 inhibition could impose growth arrest or enhance DNA damage responses. The limitation, however, is that no direct CTBP1 inhibitor has been tested in melanoma models yet. The evidence for CTBP1’s role comes from genetic experiments (shRNA or ectopic expression) showing reduced melanoma cell proliferation when *CTBP1* is knocked down (52). Without a chemical probe or drug, it’s uncertain how melanoma cells might adapt to CTBP1 inhibition. One possible issue is melanoma’s high plasticity; if CTBP1 is blocked, melanoma cells might switch to alternative pathways or even increase CTBP2 usage (though CTBP2’s role in melanoma is less studied). Another consideration is that CTBP1 is a cofactor for multiple transcription factors (e.g. it partners with ZEB1, GLI1, and others in different contexts) (136).

In melanoma, which often has diverse subclones, CTBP1’s functions could be context-dependent; in some subpopulations it might repress pro-apoptotic genes, while in others it might co-activate certain survival genes. A global CTBP1 blockade might have heterogeneous effects across tumor cells. Additionally, CTBP1’s systemic functions (metabolic regulation) could create side effects such as weight loss or hyperglycemia, which melanoma patients (often receiving immunotherapy) cannot afford. Until a CTBP1-specific agent is trialed *in vivo*, these remain open questions. Thus, while CTBP1 is a rational melanoma target on paper, the lack of preclinical therapeutic studies is a significant limitation.

Cervical Cancer-specific limitations: In cervical cancer, direct evidence implicating CTBP1 protein is scant. Instead, attention has focused on a *CTBP1*-associated lncRNA. The *CTBP1-DT* is upregulated in cervical tumors (117). *CTBP1-DT* (*CTBP1-AS2* in original manuscript) promotes cervical cancer cell proliferation, migration, and invasion, in part by acting as a sponge for miR-3163 (117). These findings identify *CTBP1-DT* (not the CTBP1 protein itself) as a promising therapeutic target in cervical cancer. The limitation here is that we still do not know if CTBP1 protein contributes to cervical cancer progression. CTBP1 may well be expressed in cervical carcinomas, but mechanistic studies are lacking. It remains an open question whether CTBP1’s oncogenic effects (e.g. repression of tumor suppressors) operate in cervical cancer cells, or if the *CTBP1-DT* lncRNA drives cancer phenotypes independently.

Without protein-level studies, drug development is in limbo. One could inhibit *CTBP1-DT*, but if CTBP1 protein is not validated as a driver, developing a CTBP1 inhibitor for cervical cancer is unwarranted. Another consideration is that HPV oncoproteins (E6/E7) rewire transcription in cervical cancer; whether CTBP1 participates in HPV-mediated pathways has not been determined. Thus, the major gaps for cervical cancer are the absence of CTBP1 protein studies and the need to clarify the relationship between *CTBP1-DT* and CTBP1. Until these are addressed, CTBP1 remains an ambiguous target in cervical cancer.

Clear Cell Renal Cell Carcinoma (ccRCC)-specific limitations: In ccRCC, a major limitation is the lack of direct protein-level evidence linking CTBP1 to tumor biology. To date, *CTBP1* expression, localization, and transcriptional activity have not been systematically interrogated in ccRCC cells or *in vivo* models. Existing data implicating the *CTBP1* locus arise largely from noncoding RNA-centered studies and bioinformatic associations, which suggest potential involvement in proliferative or immune-related programs but do not establish CTBP1 protein as a functional dependency.

Consequently, it remains unclear whether CTBP1 is required for ccRCC cell survival or tumor maintenance, or whether therapeutic targeting of CTBP1 would yield antitumor benefit. The prominence of lncRNA-mediated regulation (119), raises the possibility that locus-derived transcripts rather than CTBP1 protein itself represent the primary oncogenic drivers in this context. Moreover, given the pronounced metabolic reprogramming characteristic of ccRCC, CTBP1 inhibition could have context-dependent or tissue-specific effects, including potential disruption of renal metabolic homeostasis. Until CTBP1 is directly perturbed in ccRCC-specific experimental models, its role remains suggestive but unvalidated, limiting both mechanistic interpretation and therapeutic extrapolation.

Cholangiocarcinoma-specific limitations: Cholangiocarcinoma exemplifies a scenario where CTBP1 protein has no supporting studies, even though the CTBP1 locus yields oncogenic transcripts. To our knowledge, no published work has examined CTBP1 protein expression or function in cholangiocarcinoma tumors or cell lines. This is a critical gap; we do not know if CTBP1 contributes to cholangiocarcinogenesis. On the other hand, the LncRNA *CTBP1-DT* (*CTBP1-AS2* in original manuscript) is emerging as an important player in cholangiocarcinoma. *CTBP1-DT* is significantly overexpressed in cholangiocarcinoma tissues and cell lines; and induces G2/M cell-cycle arrest, suppresses proliferation and invasion, as well as increases apoptosis (120). These results suggest *CTBP1-DT* is an oncogenic lncRNA sustaining cholangiocarcinogenesis growth, and targeting it could be therapeutic. Notably, the complete mechanistic target of *CTBP1-DT* remains unclear. Thus, a major limitation is the entirely unexplored contribution of *CTBP1* gene to cholangiocarcinogenesis. CTBP1 protein may be irrelevant in this cancer, and the locus exerts its oncogenic impact through *CTBP1-DT* alone. These knowledge gaps must be closed before any CTBP1-centric therapy can be contemplated in cholangiocarcinogenesis.

## 7 Knowledge gaps and future directions

Despite substantial progress in defining CTBP1 as a redox-sensitive transcriptional corepressor in cancer, several conceptual and experimental gaps limit precise mechanistic attribution and therapeutic translation.

### 7.1 CTBP1-specific dependency versus CTBP-family biology

A central unresolved issue is the insufficient separation of CTBP1 from its paralog CTBP2. In multiple tumor contexts, including PDAC, AML, glioma, and ovarian cancer, functional perturbations target CTBP complexes, shared interaction motifs (e.g., PLDLS interfaces), or dual genetic reductions, without clean CTBP1-only interrogation. This conflation obscures whether CTBP1 represents an independent oncogenic driver, a context-dependent modifier, or a redundant component of a CTBP network.

Future work therefore is required for:

- Isoform-specific CRISPR KO models (*CTBP1*-only versus *CTBP2*-only).
- Rescue experiments with redox-binding–deficient or dimerization-defective CTBP1 mutants.
- Proteomic dissection of CTBP1-specific versus CTBP2-specific interactomes across tumor types.

Without this resolution, therapeutic strategies risk targeting CTBP-family biology rather than CTBP1-specific vulnerabilities.

### 7.2 Structural and redox mechanism in native tumor contexts

Although CTBP1’s NAD(H)-dependent dimerization is biochemically established, direct *in situ* evidence linking tumor NADH/NAD^+^ ratios to CTBP1 chromatin occupancy and transcriptional output remains limited. Most mechanistic models infer redox coupling rather than measuring it dynamically in tumors.

Key future directions should include:

- Chromatin occupancy mapping under controlled metabolic perturbation.
- Quantitative correlation of intracellular NADH/NAD^+^ states with CTBP1 complex assembly.
- Single-cell multi-omics integrating redox state, chromatin accessibility, and CTBP1 binding.

This would convert the redox-sensor model from conceptual framework to experimentally validated tumor biology.

### 7.3 Context-dependent oncogenic versus constraint functions

In selected cancers (e.g., melanoma), CTBP1 exhibits paradoxical behavior, potentially constraining invasive programs in certain phenotypic states. These observations suggest that CTBP1 function is contingent upon transcriptional network dominance (proliferative vs invasive states), yet systematic dissection is lacking.

Future studies should:

- Map CTBP1 target gene programs across phenotypic switching models.
- Define state-specific cofactors that determine repression output.
- Distinguish tumor-suppressive versus oncogenic CTBP1 circuits.

Defining the functional boundaries of CTBP1 across phenotypic states is essential to guide safe and context-appropriate therapeutic intervention.

### 7.4 Immune microenvironment and non-cell-autonomous effects

Emerging data link CTBP complexes to repression of antigen-presentation pathways and immune-evasion circuitry, particularly in AML. However, CTBP1-specific contributions to tumor-immune interface regulation remain incompletely defined.

Future investigations must resolve:

- Direct assessment of CTBP1 in antigen presentation, cytokine regulation, and stromal cross-talk.
- Evaluation of CTBP1 inhibition in immunocompetent models.
- Integration with checkpoint blockade or adoptive immunotherapy platforms.

Given CTBP1’s epigenetic role, immune modulation may represent a translationally relevant axis.

### 7.5 Therapeutic targetability and selectivity

Current pharmacologic approaches (except comp. 13 (109)) primarily target CTBPs dimerization or shared interaction interfaces, lacking isoform specificity and often demonstrating modest potency. Moreover, the therapeutic window remains undefined, particularly given CTBP1’s roles in normal development and tissue homeostasis.

Future development should focus on:

- Structure-guided, isoform-selective inhibitors.
- Allosteric modulators targeting redox coupling rather than global repression.
- Synthetic-lethal strategies exploiting CTBP1 dependency in metabolically rewired tumors.

Robust *in vivo* validation using conditional and inducible systems will be required to assess safety and efficacy.

- Before CTBP1 can be considered a viable therapeutic target in a specific tumor context, the following criteria should be fulfilled:
  - CTBP1 protein expression validated.
  - CTBP1-specific genetic dependency established.
  - Tumor-relevant *in vivo* efficacy demonstrated.
  - Immune-competent validation performed.
  - Therapeutic window defined.

Only tumor contexts meeting these criteria should be prioritized for CTBP1-directed therapeutic development.

### 7.6 Locus-level complexity and noncoding transcripts

The CTBP1 genomic locus encodes regulatory noncoding RNAs (*CTBP1-DT* and *CTBP1-AS*) that exert functions independent of CTBP1 protein. Several tumor associations are derived from locus-level expression analyses without protein-level validation.

Future investigations must:

- Disentangle protein-mediated versus RNA-mediated effects in detailed.
- Combine transcriptomic, proteomic, and functional perturbation approaches.
- Avoid attributing locus-driven phenotypes to CTBP1 protein without direct validation.

Further mechanistic clarification is required to determine whether *CTBP1-DT* and *CTBP1-AS* directly regulate CTBP1 protein abundance, localization, or activity, or instead function independently through distinct RNA-mediated mechanisms. Allele-specific perturbation and rescue strategies will be critical for resolving these relationships.

### 7.7 Quantitative evidence stratification across tumors

Importantly evidence strength varies widely across cancer types, from CRISPR-validated *in vivo* models to correlative expression data. A systematic framework integrating genetic perturbation, biochemical validation, *in vivo* modeling, and clinical correlation is needed to prioritize tumor contexts in which CTBP1 represents a bona fide therapeutic target.

### 7.8 Tissue-specific toxicity profiling

Comprehensive evaluation of CTBP1 inhibition in normal tissues, including bone marrow, brain, liver, and adipose tissue is currently lacking. Advanced mouse models and, where feasible, non-human primate studies will be essential to define therapeutic index and on-target toxicity.

## 8 Overall outlook

CTBP1 appears to function at an intersection of metabolism, chromatin regulation, oncogenic transcriptional control, and tumor-immune interface regulation. As a redox-sensitive corepressor, it is positioned to couple metabolic state not only to cell-intrinsic proliferation and survival programs but also to immune-relevant transcriptional circuits, including antigen presentation pathways, interferon-responsive genes, and inflammatory signaling networks within the tumor microenvironment. However, progress toward clinical translation depends on resolving isoform specificity, validating redox-dependent regulation *in vivo*, and rigorously distinguishing CTBP1-specific biology from broader CTBPs-complex effects. Addressing these gaps will determine whether CTBP1 emerges as a context-specific vulnerability influencing both tumor cell fitness and immune visibility, or remains a mechanistic contributor within a larger transcriptional repression network.

## 9 Conclusion

Across diverse cancers, CTBP1 presents as a double-edged sword: it is a compelling target due to its broad oncogenic functions (repressing tumor suppressors, altering metabolism, influencing stemness and immune evasion), yet these same features underlie the challenges of targeting it. As summarized in **Table 1** and mechanistically detailed in **Table 2**, the strength and nature of CTBP1 evidence vary substantially across tumor types, ranging from strong protein-level genetic validation with *in vivo* support to contexts dominated by CTBP-family-level or lncRNA-driven mechanisms. The evidence surveyed above highlights recurrent themes. (i), specificity is a concern, current CTBP1 inhibitors are not wholly specific and often affect the redundant family member CTBP2 (8, 121). In cancers like prostate, osteosarcoma, and others where *CTBP2* is also upregulated, a single-agent CTBP1 inhibitor may be insufficient. (ii), context matters. CTBP1’s role can vary even within the same tumor type depending on the molecular background (e.g. AR status in prostate cancer, or Wnt/APC status in colorectal cancer). This context-dependency means that blocking CTBP1 might have opposing effects in different subsets of patients. Robust biomarkers (such as a CTBP1 activation signature) will be needed to select appropriate patients, yet such markers are currently lacking. (iii), normal tissue side effects are a prominent limitation. CTBP1 participates in normal developmental and homeostatic processes; metabolism (68, 125, 134, 137), differentiation (6–8, 24, 82), immune responses (55, 93); so systemic inhibition risks toxicity in liver, adipose, bone marrow, and other systems. Strategies to mitigate this could include localized delivery (as considered in HNSCC) or intermittent dosing schedules, but these remain speculative.

**Table 2:** Tumor-specific mechanistic studies of CTBP1 across malignancies.

Tumor type	Study type	Models	Main experimental manipulation	Main conclusion
Breast cancer	Mechanistic + *in vivo* metastasis	Human breast cancer cells + orthotopic model	Protein complex/repression axis (CTBP1-ZEB1 at SREBF2 promoter); functional perturbation	CTBP1 couples metabolic inputs to EMT/invasion/metastasis via SREBF2→cholesterol→TGF-β signaling amplification.
	Metabolic syndrome + *in vivo*	High-fat diet/metabolic syndrome mouse models	*CTBP1* depletion (and metabolic syndrome context comparisons)	Systemic metabolic state (low NAD^+^/NA(H) increases CTBP1 activity and drives pro-metastatic transcriptional programs; *CTBP1* depletion abrogates these effects.
	Pharmacologic vulnerability (dimerization dependence)	Therapy-resistant/stem-like breast cancer cell states	CTBP dimerization disruption (small molecules); “genetic or pharmacologic inhibition”	NAD(H)-dependent CTBP dimerization sustains aggressive/stem-like states; dimerization disruption selectively kills resistant populations/suppresses CAIX programs.
	Promoter occupancy/transcriptional repression	Breast cancer cells; primary human cells mentioned	*CTBP1* overexpression or enforced recruitment to promoters	CTBP1 represses *BRCA1* and *CDH1* and can repress *CDKN2A*, linking CTBP1 to DNA repair and epithelial identity loss.
Prostate cancer	Functional perturbation + *in vivo*	DU145, PC3, LNCaP + xenograft/metastasis models	*CTBP1* KD	CTBP1 is required for proliferation/invasion/metastasis and contributes to radio-sensitization; *in vivo* models support dependency.
	Metabolic-context mechanistic	Mouse metabolic syndrome/high-fat diet context	*CTBP1* depletion (in metabolic syndrome-like setting)	CTBP1 connects systemic metabolic syndrome to EMT/migration programs and tumor growth.
Colorectal cancer	Genetic model context (tumor initiation/progression)	APC-related CRC contexts	Genetic perturbation context (APC loss; CTBP-linked mechanisms)	CRC literature positions CTBP1 as supporting stem-like/dedifferentiated states downstream of APC-related biology
	Protein stability/apoptosis regulation	CRC cell models	Perturbation of HERC5 E3 ligase→altered CTBP1 ubiquitination	CTBP1 ubiquitination control intersects with apoptosis regulation in CRC cells (mechanistic protein-level axis).
Lung cancer (NSCLC)	Functional perturbation + xenograft	A549, H1299 + patient-derived tumor xenografts	*CTBP1* shRNA depletion	*CTBP1* depletion reduces malignant phenotypes; xenograft evidence supports a growth-promoting role (limited overall study volume).
	Microenvironment mechanism + *in vivo* immunology-relevant	NSCLC models + syngeneic mouse experiments	*CTBP1* overexpression; CCR2 blockade or macrophage depletion (clodronate)	CTBP1 contributes NF-κB→CCL2→CCR2 macrophage recruitment/polarization; blocking CCR2/macrophages suppresses CTBP1-driven tumor growth.
	Complex disruption (partner-binding)	H1299	Cell-penetrating PLDLS-motif peptides (CPP-E1A) disrupting CTBP1 interactions (e.g., CTBP1-ZEB1)	Disrupting CTBP1 partner binding de-represses epithelial/apoptosis/repair genes (*CDH1*, *BAX*, *BRCA1*).
	lncRNA mechanism	NSCLC experimental ceRNA study	*CTBP1-DT* perturbation (ceRNA); luciferase/”rescue” described	*CTBP1-DT* promotes malignant phenotypes via miR-623→MMP3 axis.
Glioma	Complex-level metabolic/lipid program	Glioblastoma context	Repressor complex modulation (CTBPs/LSD1) via ZBTB18 framing	CTBP-associated repression integrates with lipid synthesis programs in glioblastoma (complex-based evidence).
Leukemia (AML)	Protein-protein interaction dependency	Murine hematopoietic progenitors	Disruption of AML1/MDS1/EVI1-CTBP1 interaction	CTBP1 acts as a cofactor for leukemogenic transcriptional repression; disrupting interaction impairs abnormal growth/differentiation.
	Targetability of interaction surface + *in vivo*	AML models + xenotransplant	PLDLS-competitor construct outcompeting EVI1 binding to CTBPs	Targeting CTBP interaction surface suppresses AML growth *in vitro* and in xenotransplant settings (CTBP2 emphasized, CTBP1 in same complex).
	Unbiased functional genetics + immune axis	AML	CRISPR-Cas9 screen + validation targeting CTBP1 complex	CTBP1 complex represses MHC-II; targeting restores antigen presentation and CD4^+^ T cell responses.
HNSCC	Promoter mechanism + human tissue correlation	Squamous carcinoma cells; human HNSCC tissue array	CTBP1 binding at *BRCA1* promoter (E2F4 site); NADH/hypoxia modulation	CTBP1 represses *BRCA1* in redox-sensitive manner; nuclear CTBP1 staining correlates with BRCA1 downregulation in lesions/tumors.
	Clinical correlation/bioinformatics	TCGA-based prognostic signature	Association study	*CTBP1-DT* appears in ferroptosis-related lncRNA prognostic signature; mechanism not defined.
Ovarian cancer	CTBP-family therapy-response mechanism	Ovarian cancer models	CTBP-family inhibition/depletion	CTBPs repress DR4/DR5 and gate extrinsic apoptosis; inhibition shifts toward apoptotic execution (CTBP-family, not CTBP1-specific).
	Locus product/microprotein (DDR)	Cisplatin-resistant ovarian models	*CTBP1-DT*→DDUP induction; functional linkage	*CTBP1-DT* can yield DDUP microprotein sustaining DDR and promoting platinum resistance; CTBP1-specific protein evidence remains limited.
ESCC	Strong causal genetics	Paclitaxel-resistant TE-1/PTX, KYSE-50/PTX	CRISPR/Cas9 KO of *CTBP1*	*CTBP1* loss reduces viability/clonogenicity/migration/invasion and sensitizes to paclitaxel; increases apoptosis.
	Noncoding regulator of CTBP1 protein state	ESCC	circIMMP2L axis (via HDAC1 deacetylation→ CTBP1 nuclear retention)	Noncoding RNA can lock CTBP1 into nuclear repressive state, associating with invasion/metastasis and poor outcome.
Osteosarcoma	Mechanistic complex biology	Osteosarcoma cells (as described)	CTBP1 complexes with p300/FOXO3a; HDAC1/2–IRF1; FOXM1 complex	CTBP1 scaffolds survival/immune-linked repression (*BAX*/*BIM*; interferon-stimulated genes) and contributes to chemoresistance programs.
Melanoma	Clinical correlation + mechanistic repression	Melanoma tissues + cellular context	*CTBP1* overexpression; repression targets	CTBP1 represses *p16INK4a* and *BRCA1*; inverse association between *CTBP1* overexpression and BRCA1 loss in tissues.
	Pharmacology + *in vivo*	Melanoma animal models + cellular systems	Selective CTBP1 inhibitor	A 2024 selective CTBP1 inhibitor induces cell-cycle arrest, apoptosis, EMT suppression, and inhibits primary tumor growth *in vivo*.
PDAC	*In vivo* genetics (paralog conflation explicit)	KRAS-driven PDAC model	Partial genetic ablation of CTBPs (CTBP2 haploinsufficiency with concurrent CTBP1 reduction)	CTBPs are limiting for PDAC progression/metastasis
HCC	Protein-level perturbation (cellular) + clinical correlation	HCC tissues; HepG2 and other HCC lines	*CTBP1* KD	*CTBP1* KD increases *CDH1*, reduces migration/invasion, increases apoptosis (no cell-cycle arrest); clinically *CTBP1* upregulation inversely correlates with E-cadherin.
	Microenvironment mechanism	Hypoxia context in HCC	CTBP1 inhibition (functional) in hypoxia-induced EMT	CTBP1 mediates hypoxia/NADH-driven EMT (sarcomatoid transformation) and this transition is abrogated by CTBP1 inhibition.
	Immune/ferroptosis axis (complex disruption)	HCC models	Disruption of CTBP1/HDAC complex; MAT1A rescue	CTBP1/HDAC represses MAT1A; restoring MAT1A or disrupting complex restores sensitivity to ferroptosis and CD8^+^ T cell cytotoxicity.
	lncRNA mechanism	HCC	*CTBP1-DT* KD; ceRNA described	*CTBP1-DT* promotes proliferation/migration/invasion via miRNA sponging (e.g., miR-623→CCND1; miR-195-5p→CEP55).
Gastric cancer	Clinical correlation + causal genetics	GC tissues; *in vivo* metastasis model	*CTBP1* OE; CRISPR/Cas9 KO	*CTBP1* upregulated in ~70% tumors and correlates with aggressive features; CRISPR KO reduces Stat3 phosphorylation and suppresses migration/invasion/colonies; *in vivo* metastasis constrained.
	Chemoresistance mechanism	Cisplatin-resistant AGS/DDP, HGC-27/DDP	*CTBP1* KD; RAD51 rescue	*CTBP1* depletion resensitizes to cisplatin; CTBP1 promotes resistance via RAD51 upregulation (RAD51 OE rescues).
	lncRNA mechanism	GC	*CTBP1-DT* perturbation (functional assays)	*CTBP1-DT* promotes proliferation/metastasis, inhibits apoptosis via miR-139-3p→MMP11 axis.
Cervical cancer	lncRNA-only functional mechanism	Cervical cancer cells; tissues (expression)	*CTBP1-DT* KD; ceRNA axis	CTBP1-DT KD reduces proliferation/migration/invasion and induces apoptosis; mechanism: miR-3163 sponging → ZNF217 derepression.
ccRCC	lncRNA-centered modulation of CTBP1	ccRCC cells/tissues (as described)	LINC01426 interaction with IGF2BP1→ CTBP1 abundance/activity	CTBP1 protein role not systematically mapped; evidence comes via lncRNA framework enabling CTBP1-associated repression programs.
	*CTBP1*-locus lncRNA functional	ccRCC	*CTBP1-DT* KD	*CTBP1-DT* KD reduces lipid accumulation, proliferation/migration and increases apoptosis; correlated with immune signatures/prognosis.
Cholangiocarcinoma	lncRNA-only functional mechanism	Cholangiocarcinoma cells/tumors	*CTBP1-DT* or YTHDC1 KD (m^6^A reader axis)	*CTBP1-DT* is stabilized by YTHDC1; KD induces cell-cycle arrest, reduces proliferation/invasion, promotes apoptosis; CTBP1 protein not studied.

Summary of representative mechanistic investigations of CTBP1 or CTBP-family biology across tumor types. For each malignancy, the study category (e.g., genetic, mechanistic, pharmacologic, metabolic-context, immune-axis, or lncRNA-driven), experimental models (cell lines, xenografts, syngeneic models, genetically engineered mouse models, or patient-derived samples), principal experimental manipulation (e.g., KD, CRISPR/Cas9 KO, overexpression, interaction disruption, complex modulation, or noncoding RNA perturbation), and main mechanistic conclusion are indicated. Where applicable, studies are distinguished as CTBP1-specific versus CTBP-family-level or locus-associated mechanisms. This table provides a structured overview of the experimental approaches and biological themes underpinning CTBP1-associated tumor phenotypes.

Adding further complexity, *CTBP1*-associated noncoding RNAs, including *CTBP1-AS* and *CTBP1-DT*, have emerged as functionally relevant drivers in several malignancies. **Table 1** highlights tumor contexts in which locus-associated lncRNA evidence exists in the absence of direct CTBP1 protein-level interrogation, while **Table 2** delineates the specific mechanistic frameworks through which these transcripts exert oncogenic effects. In some cancers (cervical, CCA, HNSCC), these noncoding RNAs might be more actionable targets than CTBP1 protein itself (87, 117, 120). Overall, while disrupting CTBP1’s oncogenic hub holds therapeutic promise, it is clear that one-size-fits-all approaches will not work. Future drug development must carefully address isoform redundancy, deliver agents to the right tissue compartment, and avoid collateral damage to normal cells. Only by surmounting these cancer-specific limitations can the potential of CTBP1-targeted therapy be realized.

Finally, we note that *CTBP1-DT* has been inconsistently referred to in the literature as *CTBP1-AS1* or *CTBP1-AS2*, reflecting historical naming conventions rather than a unified gene nomenclature. These alternative designations may cause confusion by suggesting antisense transcripts distinct from the bona fide divergent transcript at the *CTBP1* locus. *CTBP1-AS1*, *CTBP1-AS2*, and *PCAT10* are now recognized as aliases of *CTBP1-DT*, and accordingly, we use the HUGO Gene Nomenclature Committee (HGNC)-approved symbol *CTBP1-DT* throughout this review for clarity and consistency. To resolve naming problems in CTBP1 locus, we are suggesting following nomenclature given in **Table 3**.

**Table 3:** Standardized nomenclature and database identifiers for CTBP1 locus genes and transcripts in human and mouse.

Gene symbol	Gene name	Protein symbol	Key aliases to deprecate	HGNC ID	NCBI gene ID	Ensembl gene ID	Uniprot ID
Human							
*CTBP1*	C-terminal binding protein 1	CTBP1	BARS, CtBP1	2494	1487	ENSG00000159692	Q13363
*CTBP1-AS*	CTBP1 antisense RNA		PCAT10	48337	285463	ENSG00000280927	Not applicable
*CTBP1-DT*	CTBP1 divergent transcript		CTBP1-AS1, CTBP1-AS2, DDUP, C4orf42	28308	92070	ENSG00000196810	Not applicable
Mouse							
*Ctbp1*	C-terminal binding protein 1	Ctbp1	BARS, D4S115h, D5H4S115, 5H4S115E, CtBP3/BARS, CtBP1	Not applicable	13016	ENSMUSG00000037373	O88712
Rat							
*Ctbp1*	C-terminal binding protein 1	Ctbp1	Bars, BARS-50, CtBP3/BARS	Not applicable	29382	ENSRNOG00000005428	Q9Z2F5

Table summarizes recommended gene symbols, full gene names, legacy aliases, and corresponding database identifiers for protein-coding and noncoding transcripts at the *CTBP1* locus. Legacy names are listed for cross-referencing with prior literature. Mouse and rat ortholog information are included to facilitate cross-species comparison in experimental models.

In summary, while CTBP1 is a compelling cancer target, issues of inhibitor specificity, redundancy with CTBP2, metabolic side effects, and delivery must be addressed. The comparative evidence stratification (**Table 1**) together with the tumor-specific mechanistic mapping (**Table 2**) underscore that CTBP1 represents a robust protein-level vulnerability in a subset of malignancies, whereas in others mechanistic resolution remains limited to CtBP-family complexes or lncRNA-associated frameworks. Each tumor type may face unique hurdles (e.g. hormone signaling in breast/prostate, immune context in lung, tissue access in brain). Future drug development must carefully evaluate these limitations alongside efficacy.
